# 90,000 year-old specialised bone technology in the Aterian Middle Stone Age of North Africa

**DOI:** 10.1371/journal.pone.0202021

**Published:** 2018-10-03

**Authors:** Abdeljalil Bouzouggar, Louise T. Humphrey, Nick Barton, Simon A. Parfitt, Laine Clark Balzan, Jean-Luc Schwenninger, Mohammed Abdeljalil El Hajraoui, Roland Nespoulet, Silvia M. Bello

**Affiliations:** 1 Institut National des Sciences de l’Archéologie et du Patrimoine, “Origin and Evolution of Homo sapiens in Morocco” Research Group, Hay Riad, Madinat Al Irfane, Rabat-Instituts, Rabat, Morocco; 2 Department of Human Evolution, Max Planck Institute for Evolutionary Anthropology, Leipzig, Germany; 3 Department of Earth Sciences, The Natural History Museum, London, United Kingdom; 4 Institute of Archaeology, University of Oxford, Oxford, United Kingdom; 5 Institute of Archaeology, University College London, Gordon Square, London, United Kingdom; 6 Albert-Ludwigs-Universität Freiburg, Sedimentary Geology and Quaternary Research, Freiburg, Germany; 7 The Luminescence Dating Laboratory at the Research Laboratory for Archaeology and the History of Art, University of Oxford, Oxford, United Kingdom; 8 Muséum national d'histoire naturelle, UMR 7194 HNHP, Musée de l'Homme, Paris, France; Royal Holloway University of London, UNITED KINGDOM

## Abstract

The question of cognitive complexity in early *Homo sapiens* in North Africa is intimately tied to the emergence of the Aterian culture (~145 ka). One of the diagnostic indicators of cognitive complexity is the presence of specialised bone tools, however significant uncertainty remains over the manufacture and use of these artefacts within the Aterian techno-complex. In this paper we report on a bone artefact from Aterian Middle Stone Age (MSA) deposits in Dar es-Soltan 1 cave on the Atlantic coast of Morocco. It comes from a layer that can be securely dated to ~90 ka. The typological characteristics of this tool, which suggest its manufacture and use as a bone knife, are comparatively similar to other bone artefacts from dated Aterian levels at the nearby site of El Mnasra and significantly different from any other African MSA bone technology. The new find from Dar es-Soltan 1 cave combined with those from El Mnasra suggest the development of a bone technology unique to the Aterian.

## Introduction

North Africa yields one of the richest hominin fossil records of early *Homo sapiens* [1–3], associated with a Middle Stone Age (MSA) culture that displays some of the same characteristics as contemporaneous sub-Saharan assemblages, where early cognitive complexity is recognised [4]. These include the systematic processing and use of pigments and the production of shell bead ornaments that are argued to represent symbolically mediated behaviour. In particular, perforated marine shell beads at Blombos Cave and Sibudu in South Africa [5, 6], are remarkably similar to those recovered from sites in Morocco (El Mnasra, Contrebandiers, Taforalt, Rhafas, Bizmoune, Ifri n’Ammar [7, 8]) dated to around 82 ka or earlier [9, 10], and from Oued Djebbana in Algeria [11, 12]. Despite the presence of these objects, other diagnostic indicators of behavioural complexity, such as specialised bone tools, are only sparsely reported in the North African MSA (but see [13]). The existence of bone tools in association with the Aterian culture was reported over 60 years ago from the sites of El Mnasra and Dar es-Soltan 1 in Morocco [14]. The significance of these and other specimens has been re-evaluated in recent publications [15–18] but they have not yet been subject to a detailed study.

Here we describe a specialized bone tool discovered in association with the Aterian techno-complex from the cave site of Dar es-Soltan 1 (Morocco). The find occurs in a long cultural sequence dated by a suite of OSL age determinations providing an age of 90 ka for the bone tool bearing layer. The typological characteristics of this tool suggest a complex *chaîne opératoire* during its manufacture and its possible use as a knife. The features of this tool are similar to at least two other flat-bone artefacts from El Mnasra [14]. Together, these osseous artefacts suggest that the Aterians developed an independent North African cultural adaptation of osseous implements differing from projectile points used elsewhere in Africa during the MSA.

## Materials and methods

The bone artefact described in this paper (specimen inventory number DES1-12 N502-659) is currently housed at the Laboratory of the National Institute of Archaeological Science and Heritage, Rabat (Morocco). The permit to conduct archaeological excavations at Dar es- Soltan 1 cave was granted by the Moroccan Ministry of Culture (Authorisation numbers 676 and 677, March 14th 2012). The bone specimen is publicly deposited at the National Institute of Archaeological Science and Heritage, Rabat (Morocco) and accessible to the scientific team members and to any authorizied researcher.

### Archaeological and stratigraphical context

**Fig 1:**
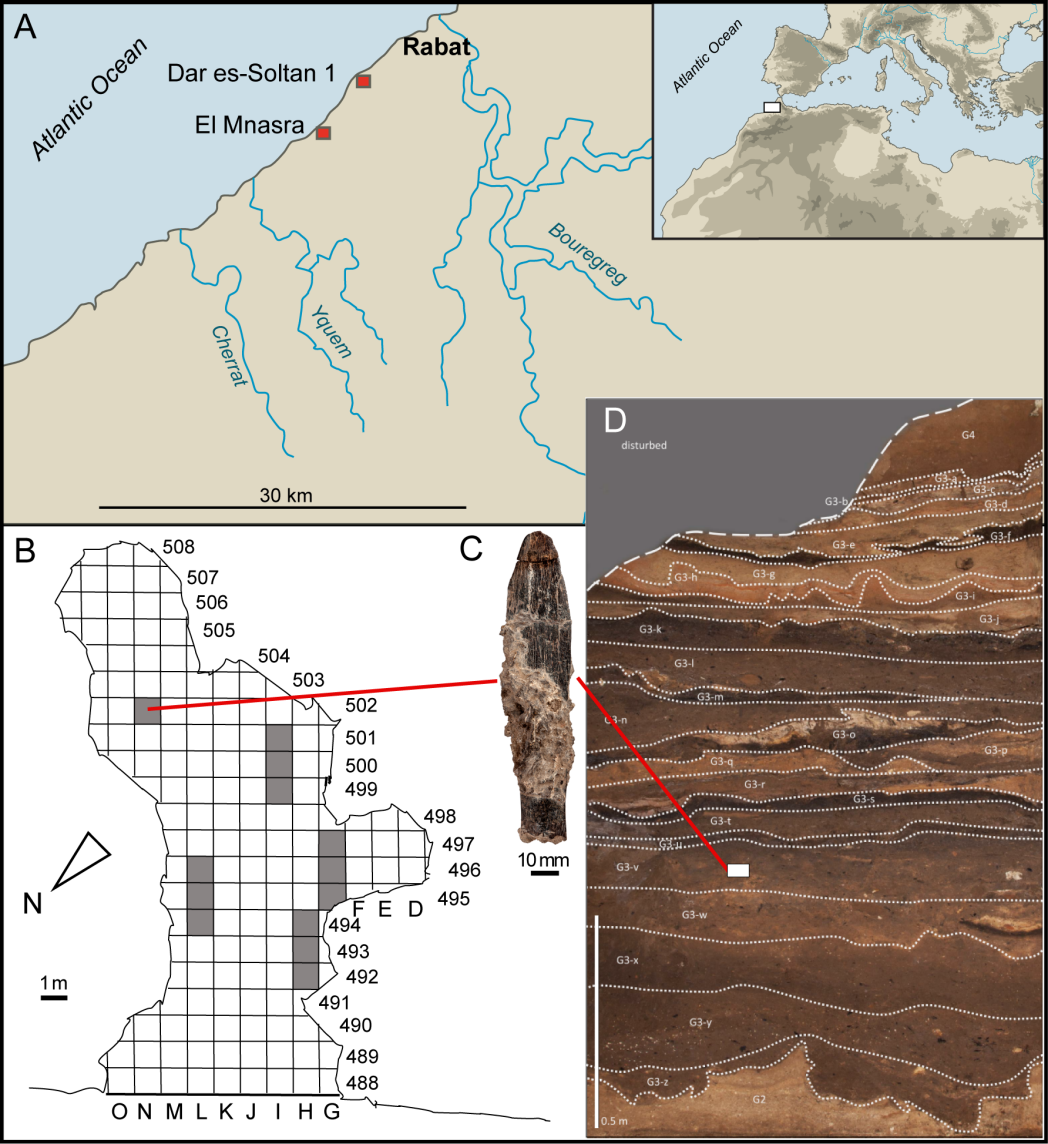
Archaeological and stratigraphical context of the bone implement from Dar es-Soltan 1. (A) Location of the Dar es-Soltan 1 and El Mnasra caves on the Atlantic coast of Morocco. Spatial (B) and stratigraphic (D) context of the bone tool (C) within Dar es-Soltan 1 cave. Shaded squares excavated in 2012. (Relief map modified from Wikimedia Creative Commons).

Dar es-Soltan 1 cave (33°58’44”N, 6°53’51”W) lies about 10 km south-west of Rabat and 260 m inland from the present Atlantic coast (Fig 1). The cave entrance faces west towards the ocean and is at an altitude of 13.52 m. The cave measures about 21 m in depth and is about 10 m at its widest point. Previous excavations by Dr. Armand Ruhlmann in 1937 and 1938 confirmed the presence of Aterian (MSA) and Iberomaurusian/Late Stone Age (LSA) layers that are rich in artefactual and organic remains. Human skeletal remains were also reported from the uppermost layers [1].

The new research program at Dar es-Soltan 1 cave was coordinate by the Moroccan Institut National des Sciences de l’Archéologie et du Patrimoine and the University of Oxford (UK), with permission from the Ministry of Culture (Kingdom of Morocco). We undertook new excavations in the cave in 2005 [19], in 2008, to collect samples for tephra analysis and to excavate a Neolithic burial in G5 and, finally, in 2012, when we excavated additional areas alongside previous excavation trenches (Fig 1B). The bone tool was found during this last season of excavation. A composite sedimentary log was recorded in various vertical segments spaced, according to clarity of exposure, along longitudinal sections left by Ruhlmann. The overall sequence has been divided into five ‘Groups’, considered to be equivalent to the grade of a geological ‘member’ and valid for all parts of the cave (Fig 1 and S1 Fig) [19].

The bone artefact described in this paper was found in unit G3.v in square N502 (Fig 1B–1D). Most of Group 3 in other parts of the cave (as reported in [2]) is almost wholly phosphatic (no HCl-reaction) and very strongly bioturbated at various scales; this has resulted in an extremely ‘fuzzy’ stratigraphy without faunal remains, that is contextually unsatisfactory, despite the common durable archaeological finds. However, in N502, both thin carbonate ash deposits and phosphatisation of the latter have contributed to bone/shell preservation. The cementation has also helped to inhibit bioturbation as did, no doubt, the relatively damp to wet condition developing in these sediments. The large capping roof-fall blocks have also inhibited later burrow penetration and have slowly added ground carbonate. Yet another factor may be that, in much of the southern side of the cave, there are deep alcoves, suitable for nesting birds and roosting bats, which would have contributed guano (aggressively acidic); in contrast, the cave walls around N502 are quite vertical, leading up to a major roof opening, perhaps being less attractive for nesting/roosting. The outcome is that bone/shell preservation in N502 is much better and the stratigraphic integrity and detail very much improved. Preliminary analysis of the faunal remains has identified auroch (*Bos primigenius*) among the large mammal material, together with smaller mammals (including *Hystrix cristata* and unidentified carnivores), reptiles (*Testudo* sp.), microfauna (S1 Table) and marine molluscs.

Only a broad level of correlation can be established between the various exposures of Group 3: units r-o (N502) correlate with G3.3 (in L500-499 for example), whilst units h-a (N502) correlate with G3.9–11. The general sedimentary sequence in the Group 3 material in N502 shows two very clear trends. First, human influence was very strong, both in terms of material input (lithics, bone, shell, charcoal, ash) and mass disturbance, at the base but waned steadily upwards, even if it never completely disappeared. Second, the area was comparatively dry at the start but became increasingly wet, with ponding and eventually current flow upwards (with much sorting of fine ash and charcoal inwards, to the east). The presence of water in the cave would therefore have acted as a limitation to the floor space available (driving actual ‘occupation’ up and westwards/outwards through time) but also as an attractor in a generally sandy landscape, where a water source may well have been useful.

**Fig 2:**
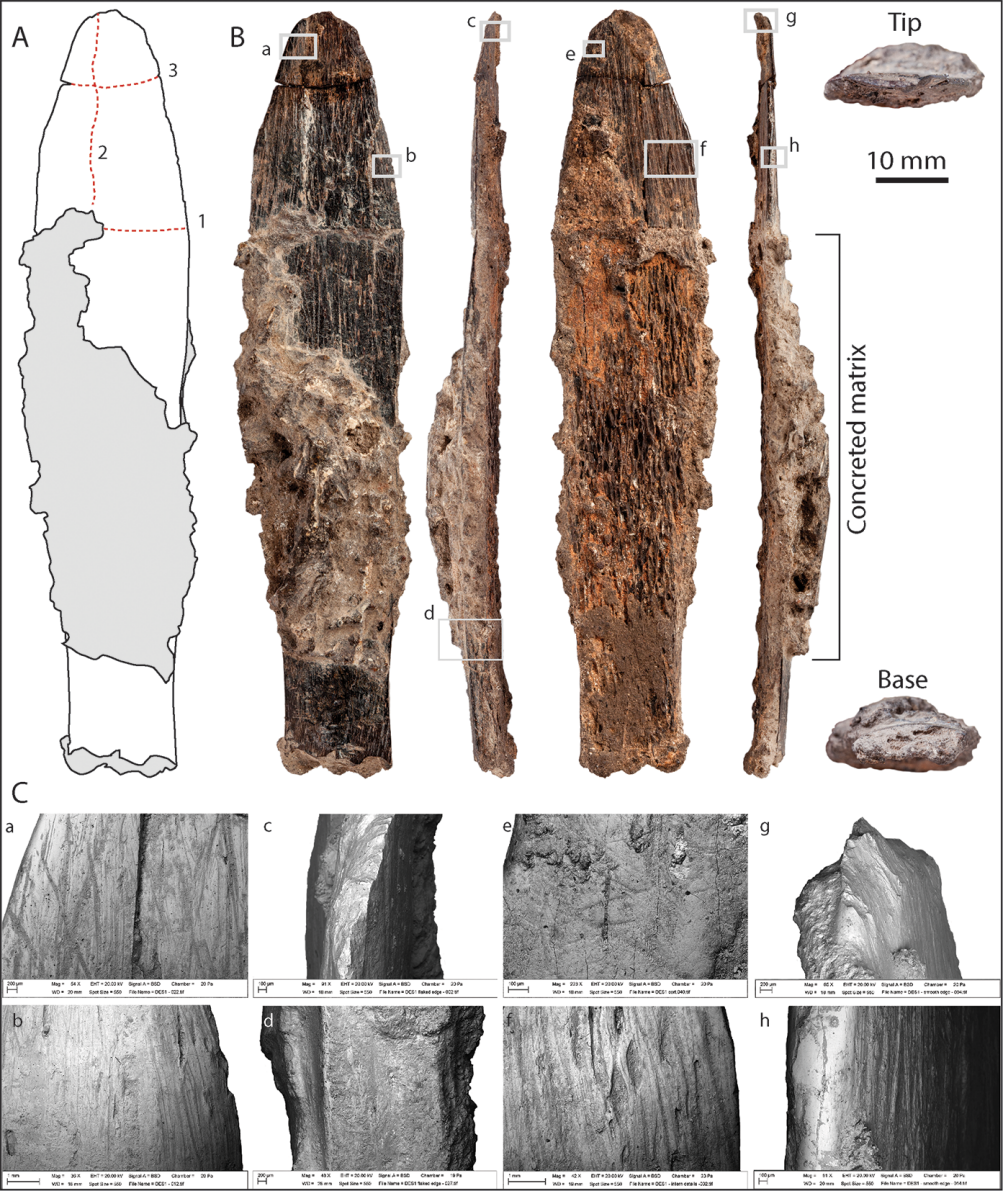
Bone tool from Dar es-Soltan 1. (A) Drawing of the tool with sequence of post-depositional breaks indicated [1–3]; (B) Photograph of the cortical side, sharp edge, trabecular side, smooth edge, tip and base of the tool. (C) SEM images detailing (a) scrape marks and polish along the smooth edge on the cortical side of the tool and (b) deep scrape marks (re-sharpening) along the sharp edge on the cortical side; (c) flaking near the tip of the tool, and (d) detail of the flat break surface near the base of the tool; (e) wear pattern near the sharp edge on the trabecular side of the tool, and (f) deep scrape-marks near the smooth edge on the trabecular side; (g) flaking close to the tip, and (h) polishing of the smooth edge of the tool.

The bone artefact was found lying in the deposit at a shallow, oblique angle such that the tip appeared first, protruding slightly through the surface. When uncovered the bone seemed to be intact, but the tip separated from the main portion of the tool during excavation. Concreted sediment embedded within the broken edges indicates that the break occurred during burial, probably as a result of trampling and sediment compression (Fig 2). The MSA lithic artefacts associated with the bone tool in square N502 include typical MSA products such as a pedunculate piece and preferential radial Levallois cores (S2 Fig) which, with other components, are common at similar levels elsewhere in the cave (S3 Fig).

The bone tool was found in a ‘sealed’ context, separated from the overlying LSA, Neolithic and Protohistoric (Group 5) horizons by an intervening MSA deposit (Group 4), which is more than 1.50 m thick. Furthermore, although 42 bone tools were discovered in the Neolithic layer B and ‘Protohistoric’ layers (layers B and A, respectively) none of these bone tools (which include a comb, two hooks, a hide-working tool, three worked teeth, including a human molar, one sawn bone, a spear-like bone, one side scraper, two perforated needles, two pins, and 28 other bone points), share any similarities with the bone tool from G3.v.

### Radiometric age model

Two sequences of OSL determinations were used to model the age of deposition of G3-v. Previously published OSL ages (Sequence 1) [19] and a new series of OSL determinations (Sequence 2) based on samples collected in 2012 from square N502 [20] were incorporated into an updated Bayesian model using OxCalv4.2. Due to the different sampling strategies used during different campaigns, the two datasets may initially be thought of as two independent stratigraphic sequences. Whilst all OSL samples within each sequence can be ordered in relation to every other sample within the same sequence, the only robust cross-correlations between the sequences are the sedimentological boundaries between Groups 2 and 3, and Groups 3 and 4. Therefore, the overall model incorporates the two stratigraphic sequences, linked only by cross-referenced probabilities calculated for these particular boundaries (S4 Fig and S2 Table). Other Group boundaries were extracted from a previous work [19] and data sequence via the ‘Probability’ command. Replicate samples OSL 5a and 5b, and OSL 48a and 48b were entered as combined likelihoods.

This model suggests that the deposition of unit G3-v occurred at approximately 90.41 ± 3.41 ka (OSL 43; S4 Fig and S2 Table). The estimate overlaps at 1 sigma uncertainty with the un-modelled OSL age of 98.3 ± 9.3 ka, but makes allowance for the slight stratigraphic age reversal between OSL 42 and OSL 43 (S4 Fig and S3 Table). The main effect of this model, however, has been to increase the precision of the OSL ages for the new samples. There is excellent agreement between the modelled ages for Sequence 1 obtained via the updated model and the results previously published [19]. Ages are nearly identical, barring slight discrepancies of less than 7 ka for samples OSL 5a/b and OSL 11. The new data indicates that Group 1 was deposited prior to ~120 ka, with subsequent group boundaries occurring at approximately 118ka, 97 ka, 73 ka, and 43 ka (S3 Table).

### The bone tool

Detailed observations of the bone tool from Dar es-Soltan 1 were conducted using standard taphonomic analyses. The state of preservation was evaluated according to bone fragmentation and soundness of cortical surface using established criteria [21–25]. Humanly-induced modifications were classified as slicing cut marks and scrape marks made during the production and resharpening of the tool edge, flaking and polishing from use [26–31]. The artefact was initially examined using a hand lens and binocular microscope. Detailed analyses of surface modifications were enhanced with observations at higher magnification using a scanning electron microscope (SEM [32, 33]). The SEM (LEO1455VP) was operated in variable pressure mode (chamber pressure 15 Pa), enabling back-scattered electron (BSE) images to be obtained without the application of a conducting layer on the specimen. SEM images were taken in order to conduct detailed analyses of surface modifications and use-wear patterns. Micro-computed tomography (micro-CT) was performed to record the surface topography, to gauge the extent of surface modifications and to resolve features in areas where the surface was obscured by concretions. The specimen was scanned using a HMX-ST CT 225 System (Metris X-Tek, Tring, UK). This scanner combines a cone beam projection system [34] with a four megapixel Perkin Elmer XRD 1621 AN3 HS detector panel; different settings were used to optimize contrast and minimize beam hardening. The long axis of the bone tool was oriented vertically with respect to the beam, thus ensuring maximum resolution whilst minimizing streak artefacts [35]. The micro-CT data were manipulated using CT-PRO software version 2.0 (Metris X-Tek) and rendered using VG Studio MAX 2.1 (Volume Graphics, Heidelberg, Germany).

**Fig 3:**
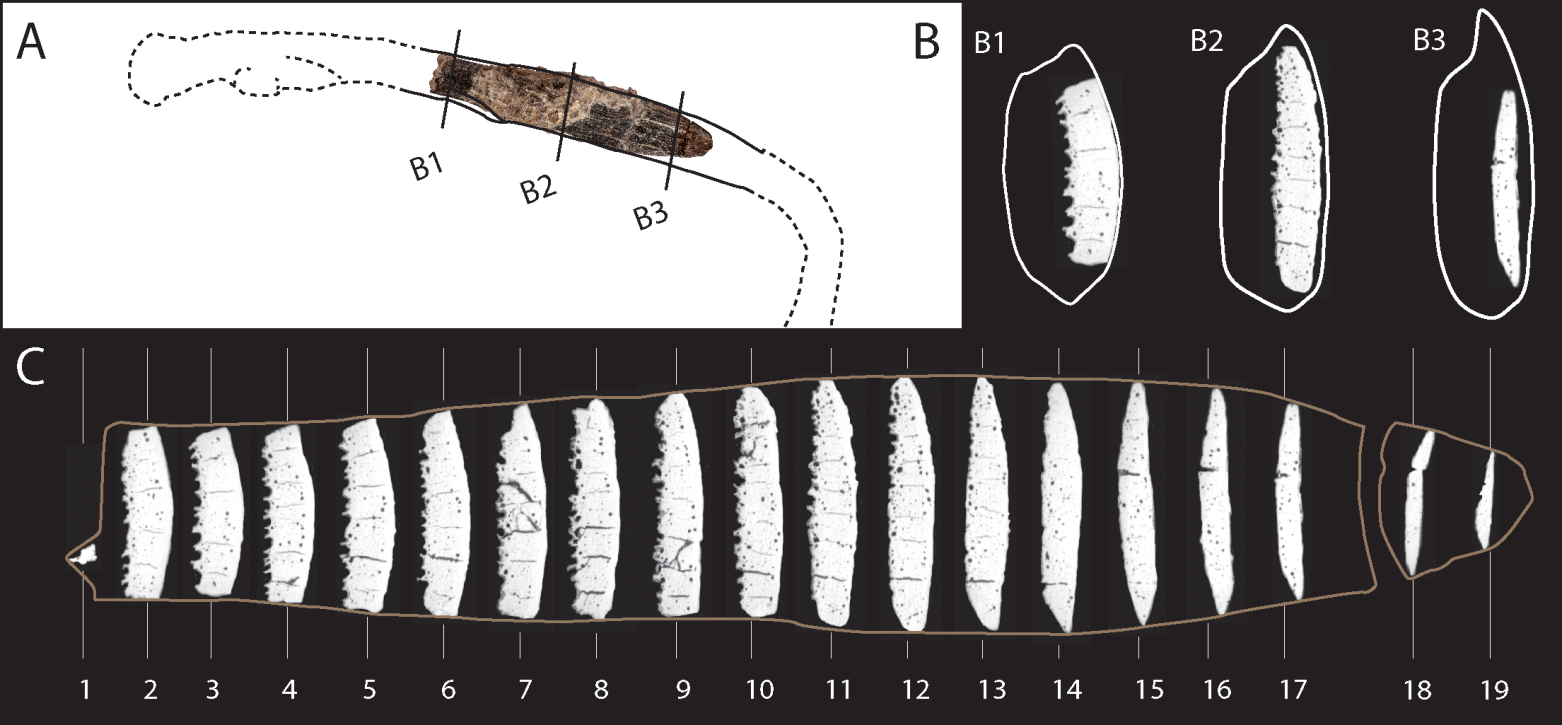
Reconstruction and micro-CT sections of the bone tool from Dar es-Soltan 1. (A) Reconstruction of the portion of a large mammal rib used in the manufacture of the bone implement from Dar es-Soltan 1. (B) Micro-CT scan sections close to the base (B1), the middle (B2) and the tip (B3) of the tool (white outlines show the reconstructed cross-section of the rib). (C) Sequence of 19 micro-CT scan sections superimposed on an outline of the bone tool with sediment digitally removed (C2).

The Dar es-Soltan 1 bone tool was produced on an oblong ‘plaque’ of bone that is gently curved both longitudinally and transversely (Fig 2). The dimensions, cortical thickness and overall morphology of the trabeculae suggest it is formed from a portion of rib shaft, consistent in size with a large mammal, probably from a bovid (Fig 3A).

The reconstructed length of the bone tool is 122 mm: the length of the main piece is 111 mm and the smaller piece is 12 mm long. We refer to the smaller piece as the ‘tip’ (distal end), and the other end is defined as the ‘base’ (proximal end) of the implement. The width of the tool varies from 18 mm to 27.5 mm along its length, resulting in a ‘fish-shaped’ outline with tapered edges converging towards the rounded tip (Fig 2). The maximum thickness of the piece at the base is 4.7 mm; scraping has reduced the thickness of the cortical bone to ~1.2 mm at 5 mm from the tip.

The tool presents at least three flexion breaks and longitudinal internal cracks (Fig 2A). The first flexion break (1 in Fig 2A) occurred transversely across the specimen at 17 to 18.5 mm from the tip end. This resulted in two pieces now held together by sediment; the break caused a slight deflection of the longitudinal profile. The shorter fragment then broke longitudinally (2 in Fig 2A) and the narrower part was also bent, causing further distortion. Finally, a second transverse break (3 in Fig 2A) separated the tip from the middle section. The base is broken transversely probably during manufacture and shaping of the tool. This sequence of the breaks and pattern of distortion would suggest that the tool was discarded in one piece and that the three breaks towards the tip only occurred after deposition. No cut marks that can be unambiguously related to defleshing or skinning were observed on the rib fragment; however any such marks could have been erased by repeated scraping of the bone surface associated with the shaping of the tool (see below). Similarly, no traces from carnivore chewing or weathering were observed.

**Fig 4:**
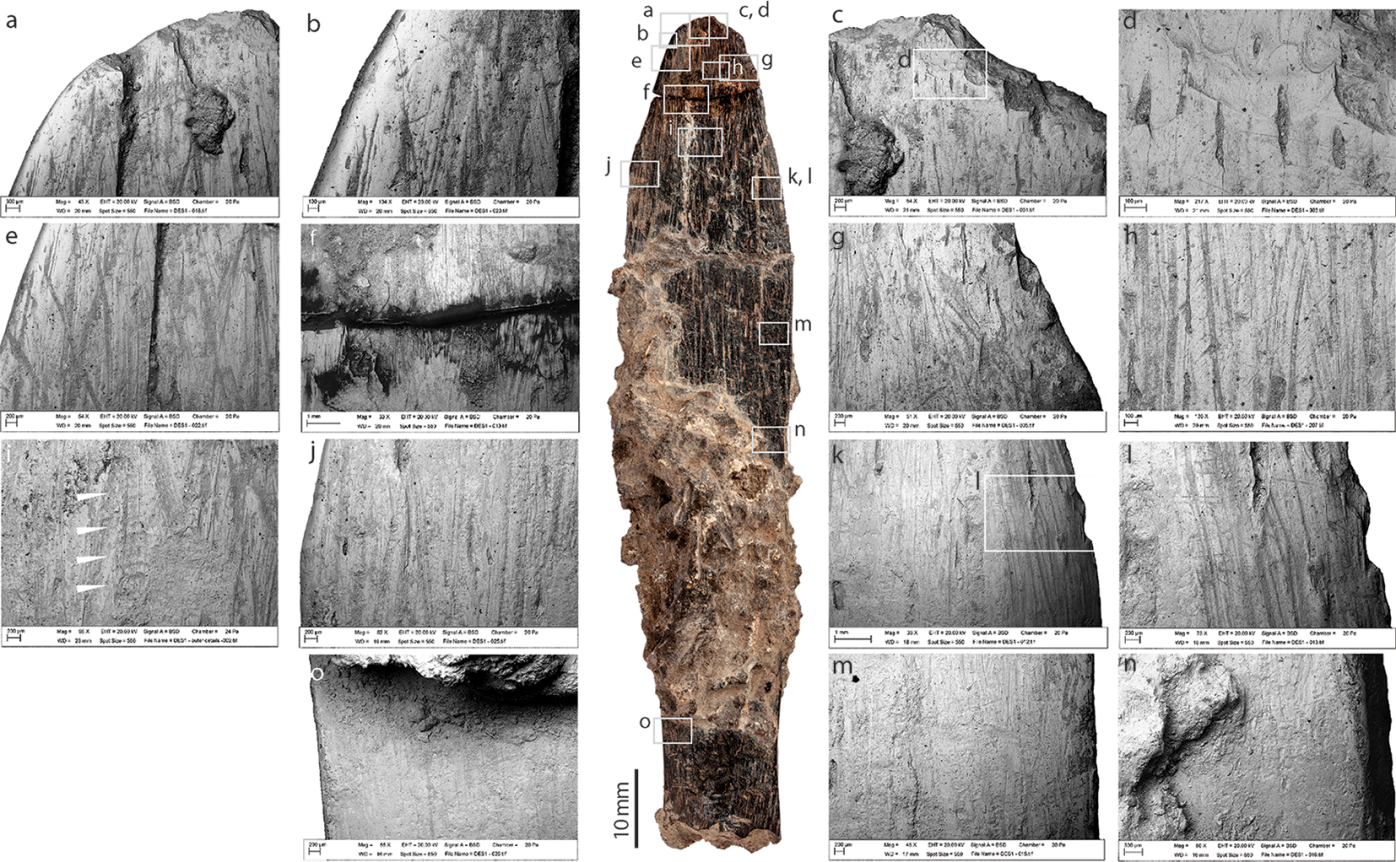
Cortical side of the bone tool from Dar es-Soltan 1. Photo and details of bone surface modifications. (SEM images, a to o).

Part of the tool is obscured by sediment still cemented onto the central area on both sides of the specimen (Fig 2). This concretion matches the carbonates found in same sedimentary horizon. After testing the feasibility of removing the adhering sediment from a small area at the proximal end, it was decided that further mechanical preparation would result in damage to the surface (Figs 2B and 4O), and therefore the remaining sediment was left untouched.

The manufacture of the bone tool followed a succession of precise actions. The rib was first modified to reduce its length and bisected longitudinally along the cranial and caudal edges to produce a shorter and narrower ‘blank’. Adherent sediment along with subsequent scarping and use wear (Fig 2) make it difficult to determine whether the breaks took place when the bone was fresh or dry, or whether cutting, chipping or abrasion were used to shape the blank. However, deliberate shaping of the rib is clearer on the sides of the implement, where the cranial and caudal edges were modified to create more or less straight sides (Fig 3, micro-CT scans 2 to 9). The removal of the cranial and caudal margins of the rib would have been undertaken to facilitate longitudinal splitting of the rib into two halves. The longitudinal division of the rib into two halves results in two surfaces: one cortical (we will refer to this as the ‘cortical side’), and the other mainly composed of trabecular bone (‘trabecular side'; Fig 2). One edge is much sharper due to further thinning of the cortical and trabecular surface by scraping and flaking towards the tip (‘sharp edge’), whereas the other edge is more rounded with polishing that obliterates the scraping marks along much of its length (‘smooth edge’).

#### Cortical side

Most of the cortical side is modified by long longitudinal scrape marks with a main orientation parallel to the long axis of the bone. At higher magnification, the scrape marks show broad zones of micro-striations (Fig 4A–4J and 4H–4L), which are typical of incisions produced by a stone tool used in a scraping action (e.g. [36, 37]). On the cortical surface, scrape marks extend for approximately 60 mm from the tip of the tool to where they become hidden by the concretion; scrape marks appear to be on the basal exposed part of the cortical surface. Scrape marks in proximity of the edges and the tip (Fig 4G, 4K and 4L) converge towards the midline of the tool. They are particularly deep toward the tip of the tool, where more intensive and concentrated scraping has removed the cortical surface and exposed the underlying osteons (Fig 4C, 4D, 4G, 4K and 4L). Towards the distal and mid portions of the implement, (Fig 4I), scraping has produced a marks characterised by internal micro-‘waves’ perpendicular to the long axis of the scrape marks. These ‘chattermarks’ were produced when the stone tool juddered across the bone surface due to the great pressure used during the scraping action. Fine overlapping striations perpendicular and oblique to the main axis of the tool, are visible close to the sharp edge of the tip of the tool (Fig 4D, 4G, 4H and 4L); these marks are interpreted as use-wear. In contrast, towards the smooth edge of the tool, more extensive areas of polishing have obliterated the scrape marks (Fig 4A, 4B and 4E).

#### Trabecular side

**Fig 5:**
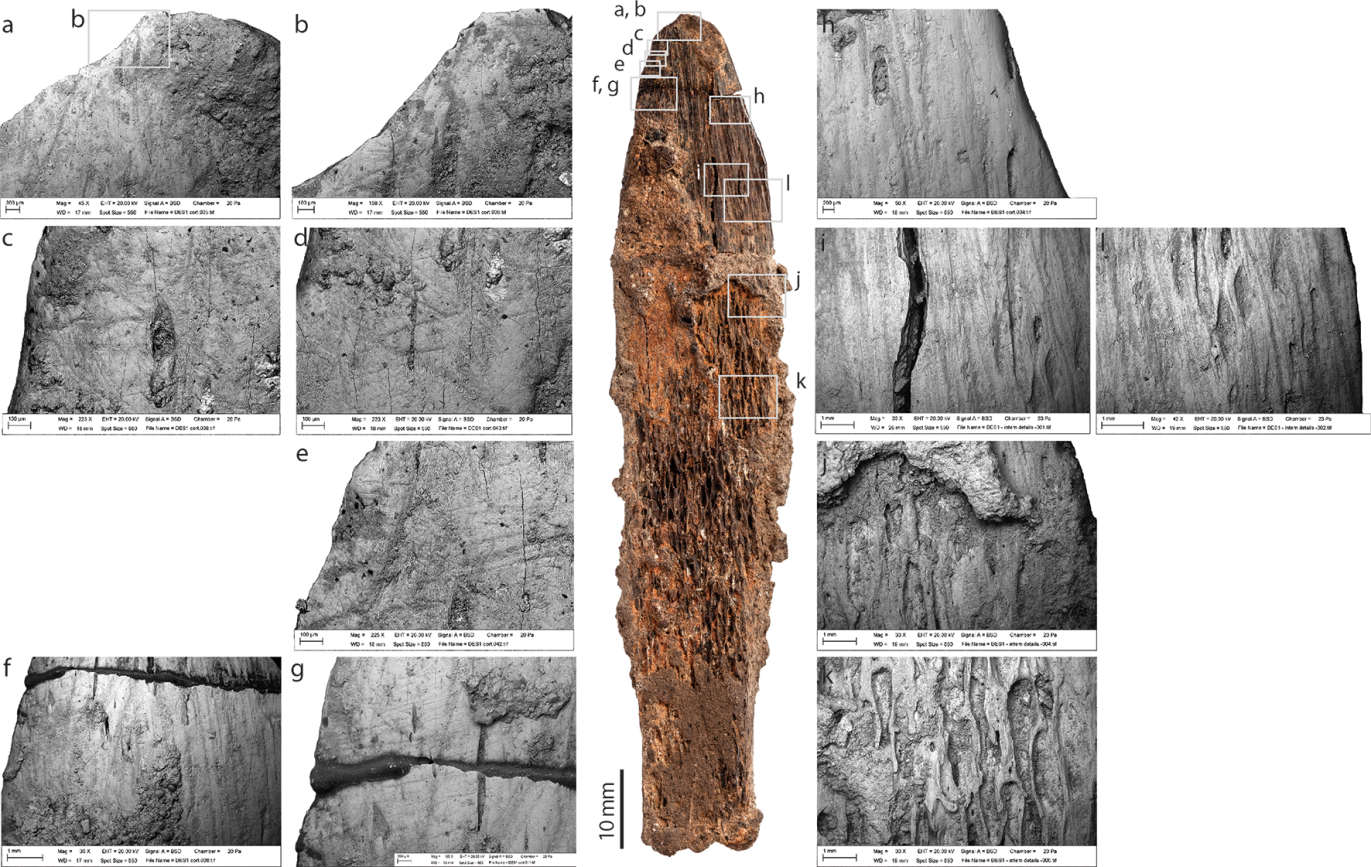
Trabecular side of the bone tool from Dar es-Soltan 1. Photo and details of bone surface modifications. (SEM images, a to l).

On the trabecular side, the scrape marks are found primarily towards the tip of the tool and appear to stop around 35 mm from the tip (Fig 5I and 5J). The scrape marks are generally parallel to each other and more intense scraping occurred towards the tip. Here, scraping has removed trabecular bone and exposed denser cortical bone beneath (Fig 5A–5I). These modifications suggest that scraping was an integral component in the thinning and shaping of the specimen (also observed at other MSA sites) [38, 39]. Use-wear in the form of fine overlapping striations perpendicular and oblique to the main axis of the tool, is visible on the trabecular surface close to the sharp edge of the tip of the implement (Fig 5A–5G). Use-wear is more intense close to the tip of the tool and partially masks the earlier scrape marks.

#### Sharp edge

**Fig 6:**
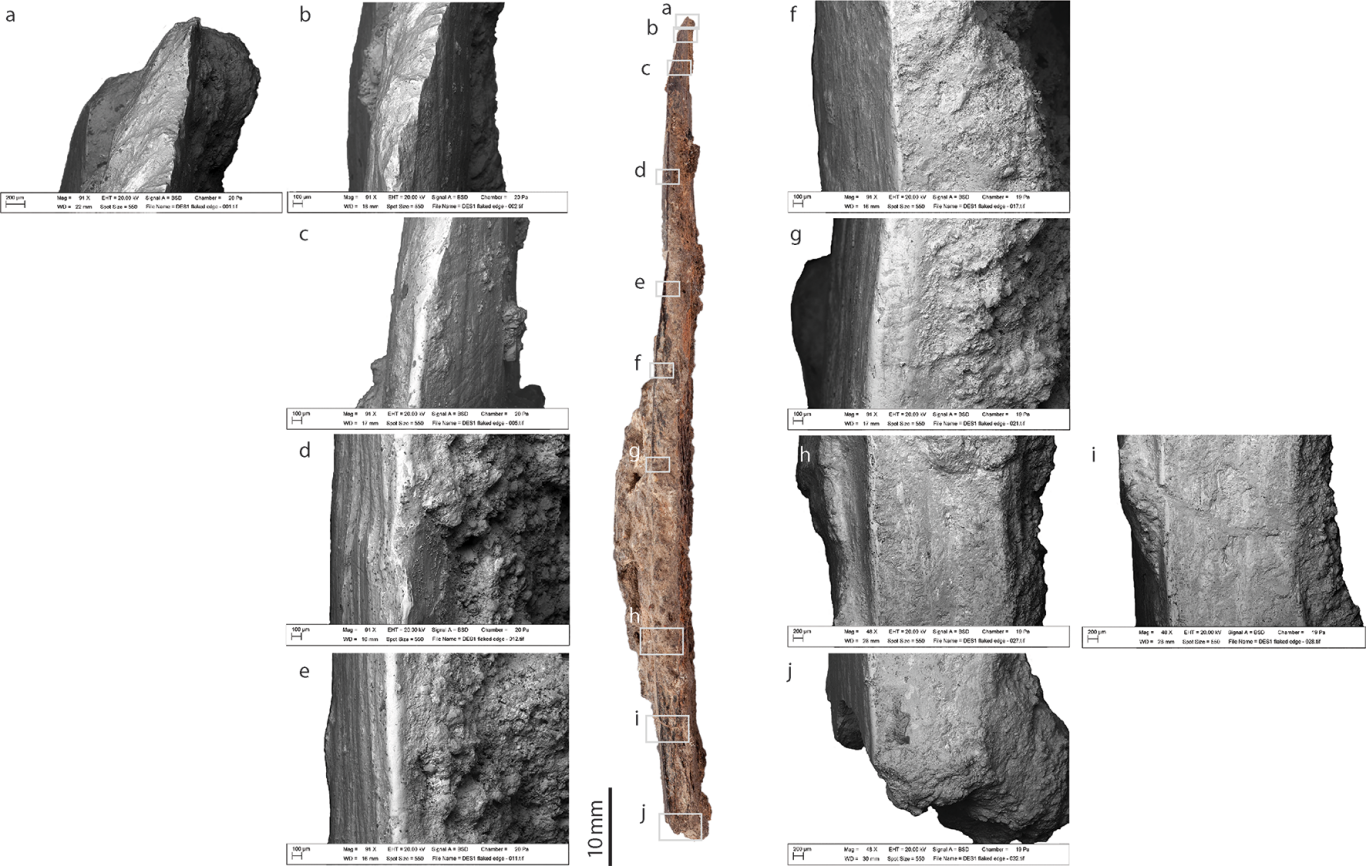
Sharp edge of the bone tool from Dar es-Soltan 1. Photo and details of bone surface modifications. (SEM images, a to j)

The tip of the implement and the edge near the tip are sharp due to repeated thinning of the cortical and trabecular surface by scraping and flaking (Fig 6A–6C). The flaking of the cortical bone could have resulted from incidental damage during use. The edge becomes smoother and polished in the basal and mid sections of the implement (Fig 6D, 6E and 6G). A visible straight surface of the edge is particularly visible toward the mid-basal portion of the tool (Fig 6F–6J).

#### Smooth edge

**Fig 7:**
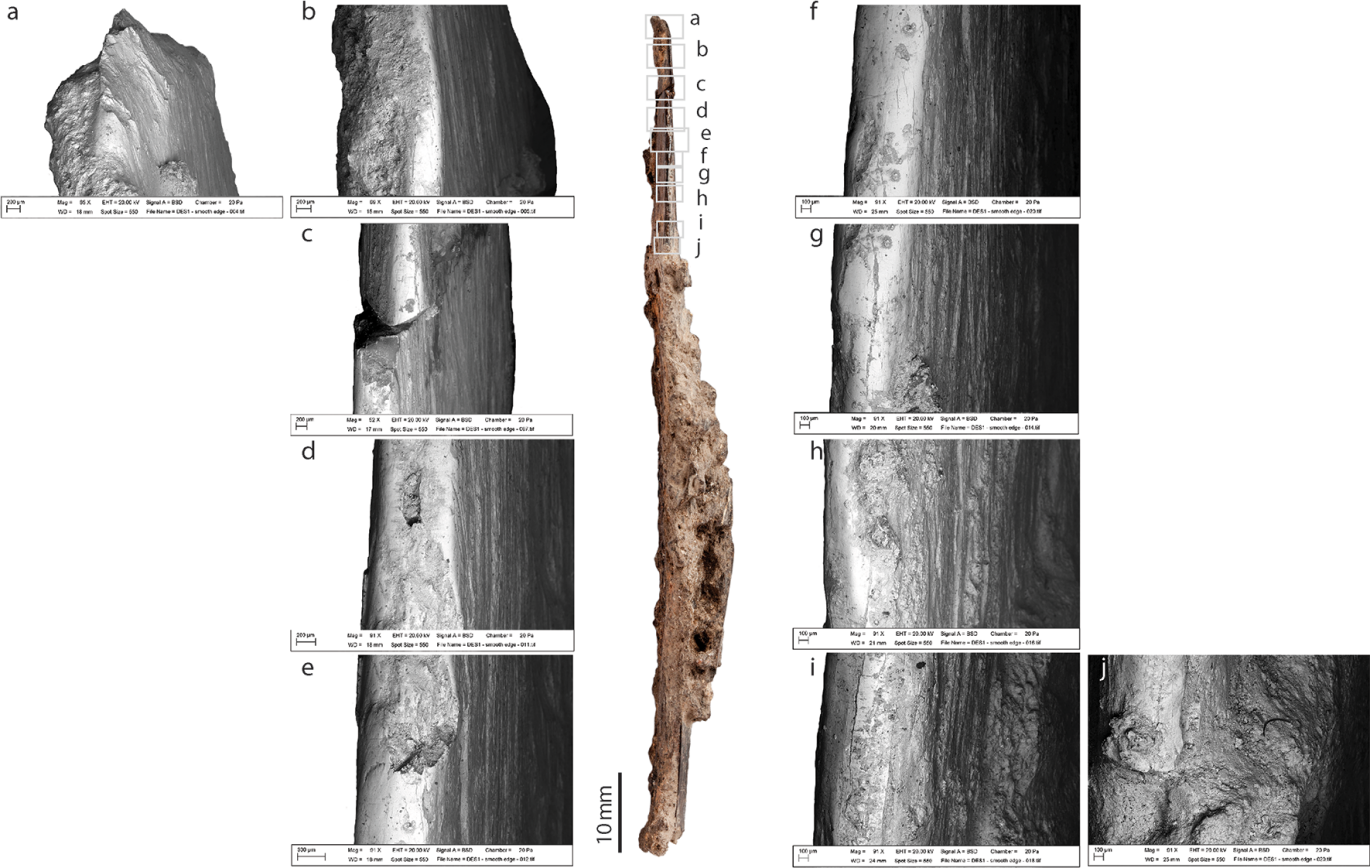
Smooth edge of the bone tool from Dar es-Soltan 1. Photo and details of bone surface modifications. (SEM images, a to j)

While the tip of the bone knife is sharp (Fig 7A), this sharpness disappears almost immediately along the smooth edge, substituted by a smooth polished surface (Fig 7B–7J). Wear pattern in the form of pitting and scratches appears only in one area of this edge (Fig 7).

Use-wear is generally limited and it manifests in the form of fine overlapping striations perpendicular and oblique to the main axis of the tool, visible on the trabecular, and, in fewer number, on the cortical surfaces close to the flaked edge of the tip of the tool (Figs 2Cb and f, 4A, 4B, 4K, 4L and 5A–5G). The use-wear striations are absent towards the ‘smooth edge’ of the tool, where they are substituted by large areas of polishing on both the trabecular and cortical sides. The polishing pattern, which masks the scrape marks in this area, is comparable to the effect of experimental handling of osseous material [40]. A hypothesis, therefore, could be that the bone tool was handled with one or more fingers firmly pressed against the smooth edge in proximity of the tip of the tool while using the distal portion of the flaked edge for cutting. This type of handling would be more typical of a knife, hence we suggest this tool was a bone knife, with multiple possible applications. The limited wear pattern, however, suggests that this particular knife was typically used for light duty work, possibly cutting of soft material [41]. The presence of flaking and fine striations also suggests occasional contact with more resilient material.

## Discussion and conclusions

**Fig 8:**
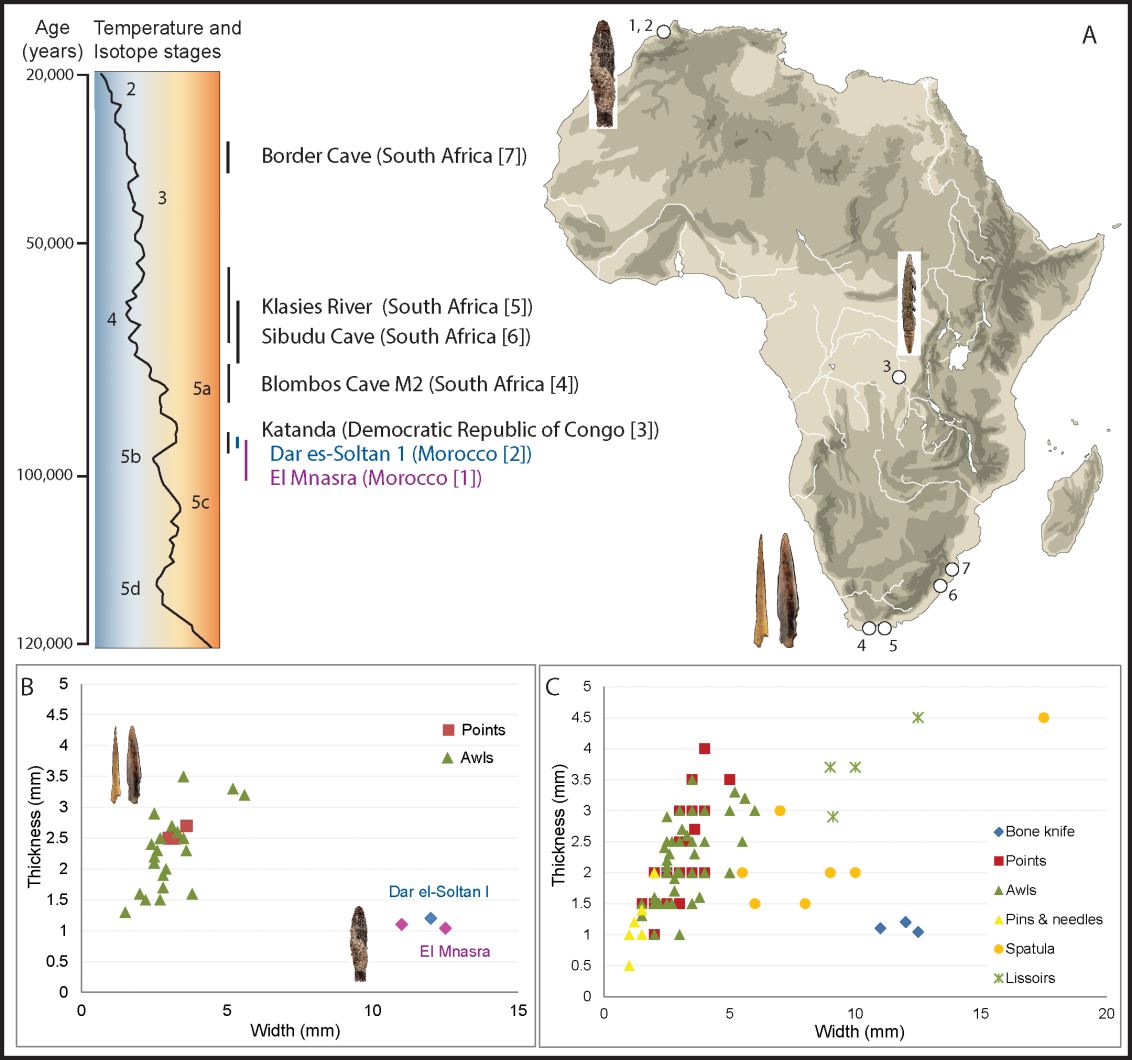
Comparison of the bone tools from Dar es-Soltan 1 and El Mnasra with other types of bone implements. (A) Temporal and geographical distribution of the African Middle Stone Age (MSA) sites discussed in the text. (B) Measures of thickness and width of an implement at 5mm from the tip of bone tools for African MSA sites. (C) Measurements of thickness and width at 5mm from the tip of compared implements. Details of measurements and sources are presented in SI4. (Images of Katanda and Blombos bone tools have been modified from photos, credit to the Smithsonian National Museum of Natural History, http://humanorigins.si.edu/evidence/behavior/making-clothing/bone-awls).

One of the diagnostic indicators of cognitive complexity is the presence of specialised or formal bone tools [42, 43], defined as functional artefacts shaped and modified using techniques such as scraping, grinding, grooving and polishing [38]. Specialised tools were previously only known from the Old World Upper Palaeolithic with few examples recently reported for the Middle Palaeolithic of Europe [44]. In Africa, their antiquity extends back into the Middle Stone Age [38, 42, 45] (Fig 8A).

The oldest specialised bone tools so far known from sub-Saharan Africa come from Katanda (Democratic Republic of Congo) and are dated to ~90 ka [42]. These examples include delicately worked barbed harpoons and are unlike the artefacts from Morocco. Elsewhere, in South Africa, pointed bone tools have been recovered in the Stillbay (SB) layers of Blombos Cave (M2 phase) dating to between ~84 and 76 ka [46–48], while at Sibudu Cave a range of bone tool types has been recorded including examples from pre-SB layers (>72 ka) [6, 38]. Examples from slightly younger contexts have been recorded at Border Cave [49]. With the exception of the specialized bone tools from Sibudu Cave and Katanda, these early finds are restricted to pointed forms.

**Fig 9:**
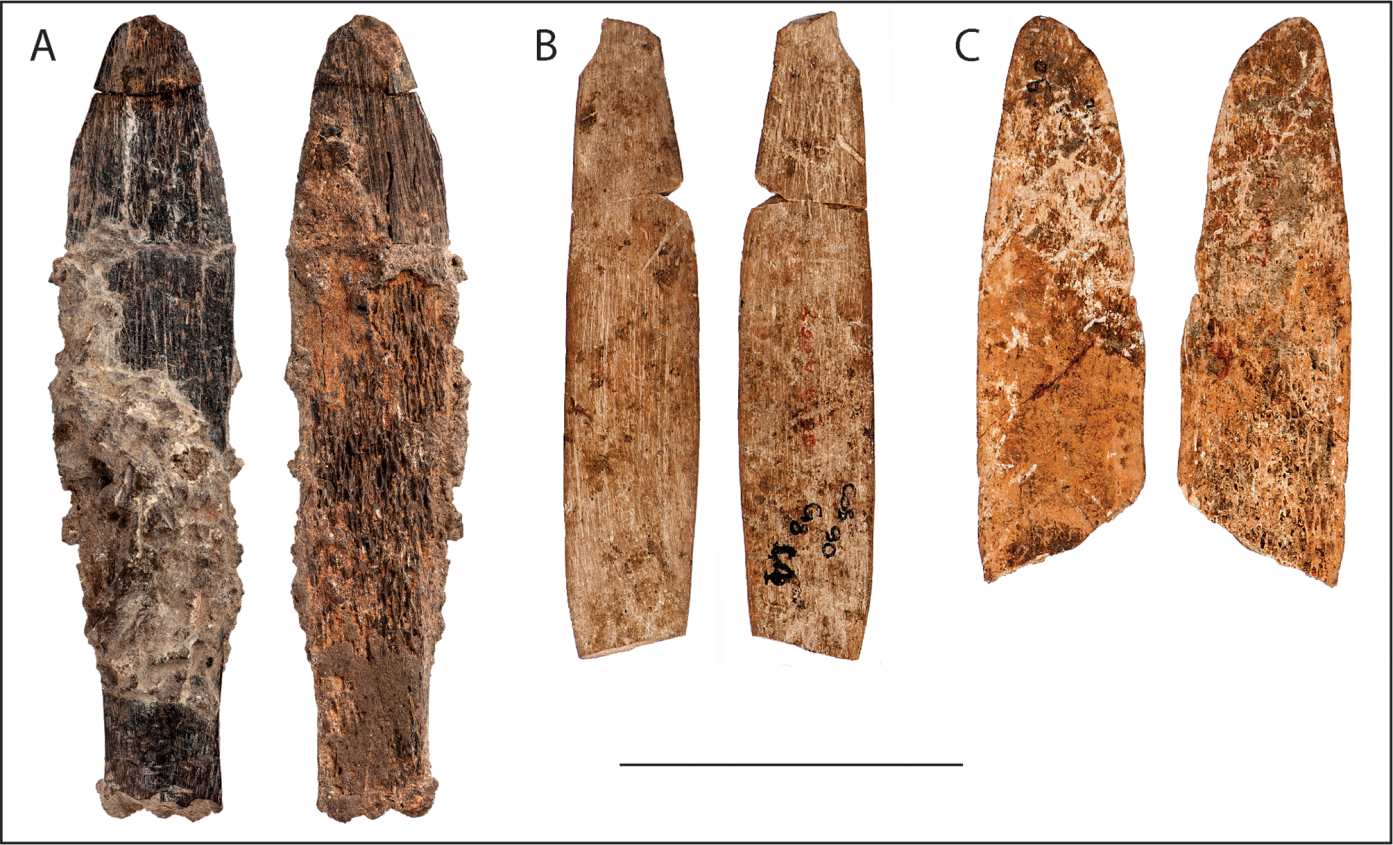
Middle Stone Age bone knives from Dar es-Soltan 1 and El Mnasra. (A) Dar es-Soltan 1 bone knife; El Mnasra (B) refit of E9-233 with E9-229 and (C) D11-T194. Cortical and trabecular surfaces. Scale = 50 mm. (Relief map modified from Wikimedia Creative Commons).

With a date of approximately 90 ka, the tool from Dar es-Soltan 1 represents the oldest specialised bone tool yet found in a well-dated context that is clearly associated with the Aterian culture. Eleven osseous artefacts have previously been described from Aterian deposits from El Mnasra cave, which have been dated by OSL to approximately 111–106 ka [17, 18] and by ESR-U/Th to approximately 89-67ka [15, 50]. Two of these bone objects (D11-T194 and refit of E9-233 with E9-229) have characteristics similar to the Dar es-Soltan 1 bone knife. Both were produced on ribs of large mammals that were modified in a way comparable to the *chaîne opératoire* adopted for the Dar es-Soltan 1 bone tool. One of the tools (refit of E9-233 with E9-229) is produced on a rib of a mammal of smaller dimensions than Dar es-Soltan 1. It has not been split into two halves, as with the Dar es-Soltan 1 knife, but its bone surfaces were also thinned by repeated scraping. D11-T194 has overall dimensions comparable with the Dar es-Soltan 1 knife, and it was split longitudinally into two halves in a similar way. Both the cortical and trabecular surfaces were further thinned by repeated scraping of the surface to produce an elongated slender object [14, 15] (Fig 9).

The measurements of the tips of the El Mnasra bone tools match those of the tip of the bone knife from Dar es-Soltan 1, confirming that not only the manufacture but also the dimensions of these artefacts are remarkably similar (Fig 8B and S3 Table). We suggest that these bone tools from El Mnasra and Dar es-Soltan 1 have characteristics typical of bone knives and that the use-wear present on the Dar es-Soltan 1 knife indicates a possible use for cutting soft material.

The production of this new type of tool may be related to changes in the pattern of resource exploitation by humans. The period around 90 ka corresponds to an increase in the availability of marine resources, and it is possible that new tools were necessary to accomplish new tasks. Further archaeological discoveries and experimental work should establish whether there is a direct association between these implements and the exploitation of marine resources.

Regardless of how the bone knives from Dar es-Soltan 1 and El Mnasra were used, the complex and controlled sequence of actions involved in their manufacture is so far unique in the Aterian archaeological record. Equally unique are their shape and dimensions, which separate them from other formal bone tools found in MSA Sub-Saharan African sites (e.g. bone points and awls) as well as spatulae and lissoirs from Upper and Middle Palaeolithic European sites (Fig 8C and S3 File). Such bone technology is another distinctive characteristic associated with the precocious appearance of symbolic artefacts and suggest the emergence of an independent modern techno-complex unique to the Aterian culture in North Africa around ~100 ka.

## Supporting information

S1 Fig Dar es-Soltan 1 cave,square N502. Stratigraphic and archaeological contexts () and OSL dates published in [1] (Sequence 1). A second set of OSL dates (Sequence 2) was obtained from square N502 in 2012.
(TIF)

S2 Fig Example of lithic artefacts from archaeological units in G 2, 3 and 4 attributed to the MSA/MP at Dar es-Soltan I cave. (TIF)

S3 Fig Bordian cumulative graph. The horizontal axis refers to the list of Bordian types and the vertical axis represents the cumulative sum of the percentages of each type.
(TIF)

S4 Fig OSL dates model structure and results for Bayesian analysis incorporating previously published ages [1: Sequence 1] and new results (Sequence 2). OSL likelihoods (dark grey) and modelled ages (light grey) comprising each sequence are displayed in stratigraphic order; OSL43 from the bone tool bearing layer G3-v has been highlighted in green. The modelled cross-correlations (Group 3 boundaries) are shown in orange, and all other group boundaries in blue. Age ranges for sedimentary Groups 1–5 are indicated at the base of the figure.
(TIF)

S1 File Archaeological context. (DOCX)

S2 File OSL dates. (DOCX)

S3 File Measurements of specialised bone tools. (DOCX)

S1 Table Small mammals from Dar es-Soltan 1. Small mammal taxa identified from sieved samples, together with the number of isolated teeth, mandibles and maxillae assigned to each (NISP) and the minimum number of individuals (MNI) represented.
(DOCX)

S2 Table Code for updated Bayesian model of Dar es-Soltan I, implemented in OxCal 4.2. (DOCX)

S3 Table Posterior probabilities for OSL ages, sequence boundaries, and probabilities representing sedimentological group transitions. (DOCX)
