# Supplementary figures and images for: 90,000 year-old specialised bone technology in the Aterian Middle Stone Age of North Africa
Source: PLoS One. 2018 Oct 3;13(10):e0202021. doi: 10.1371/journal.pone.0202021 (PMC6169849; doi:10.1371/journal.pone.0202021)

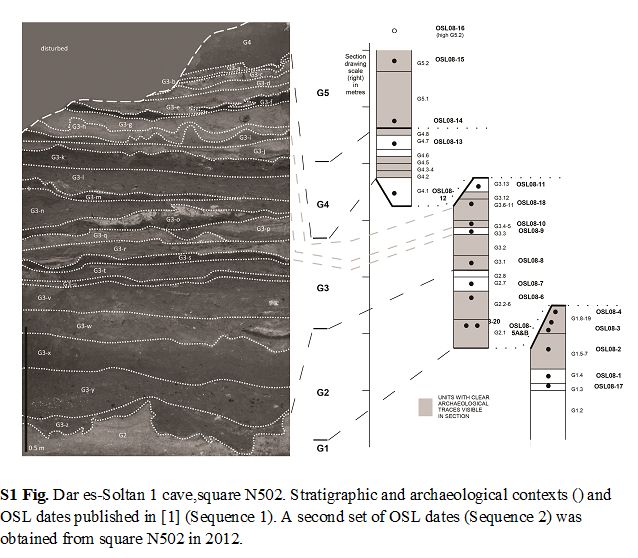

Supplement: S1 Fig — Stratigraphic and archaeological contexts () and OSL dates published in [1] (Sequence 1). A second set of OSL dates (Sequence 2) was obtained from square N502 in 2012. (TIF) [file pone.0202021.s001.tif]

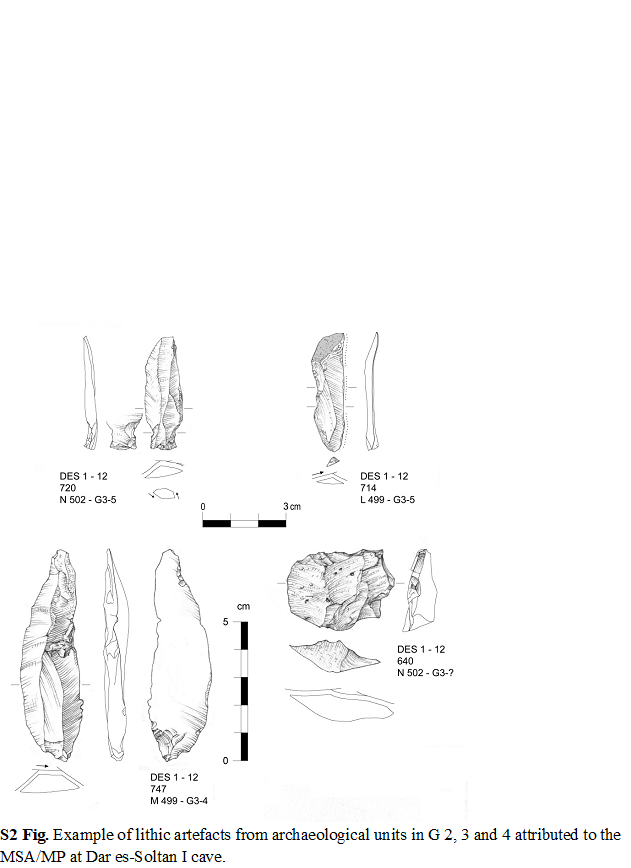

Supplement: S2 Fig — (TIF) [file pone.0202021.s002.tif]

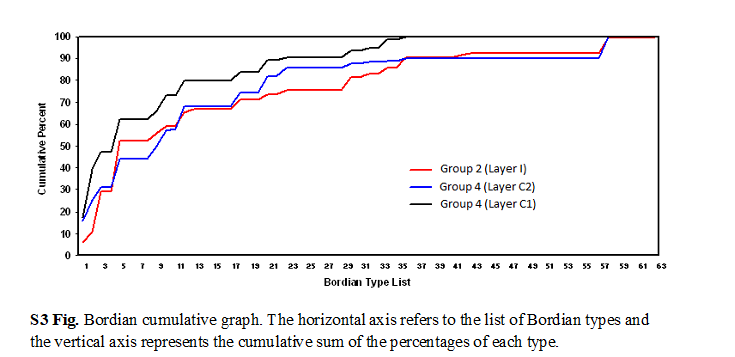

Supplement: S3 Fig — The horizontal axis refers to the list of Bordian types and the vertical axis represents the cumulative sum of the percentages of each type. (TIF) [file pone.0202021.s003.tif]

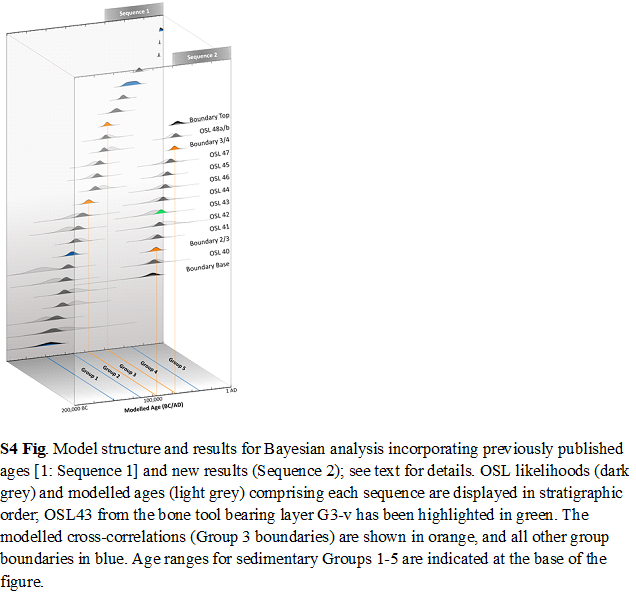

Supplement: S4 Fig — OSL likelihoods (dark grey) and modelled ages (light grey) comprising each sequence are displayed in stratigraphic order; OSL43 from the bone tool bearing layer G3-v has been highlighted in green. The modelled cross-correlations (Group 3 boundaries) are shown in orange, and all other group boundaries in blue. Age ranges for sedimentary Groups 1–5 are indicated at the base of the figure. (TIF) [file pone.0202021.s004.tif]
