# Supplementary material for: 90,000 year-old specialised bone technology in the Aterian Middle Stone Age of North Africa
Source: PLoS One. 2018 Oct 3;13(10):e0202021. doi: 10.1371/journal.pone.0202021 (PMC6169849; doi:10.1371/journal.pone.0202021)
Supplement: S1 File — (DOCX) [file pone.0202021.s005.docx]

**90,000 year-old specialised bone technology in the Aterian Middle Stone Age of North Africa**

Abdeljalil Bouzouggar, Louise T. Humphrey, Nick Barton, Simon A. Parfitt, Laine Clark Balzan, Jean-Luc Schwenninger, Mohammed Abdeljalil El Hajraoui, Roland Nespoulet, Silvia M. Bello

**S1 File**

**Archaeological context**

**Lithostratigraphic descriptions**

The following sedimentary log was recorded forsquare N502.The sequence has been divided into five ‘Groups’ (broadly of ‘member’ lithostratigraphic rank), based upon significant sedimentological differences and major erosional events or unconformities (S1 Fig).

G2 Top [200-170]; sands, good horizontal bedding; strong phosphate content (diffused and nodular, weakly phosphate-cemented); sterile of finds; very pale brown 10YR 8/4 with ‘ginger’ streaks; upper boundary extremely sharp and irregular, at scales predominantly of 1cm and 20 cm (bioturbation, possibly including human activity. [OSL date = OSL DES12-40, X4147, <825>]

G3-z [170-165]; mixed interface material, wholly chaotic, patchy and variable; some finds of lithics, bones, charcoal.

G3-y [165-156]; silty fine sands with white phosphate specks, small pebbles, only local stratification surviving; large charcoal fragments, lithics and chips, bone, microfauna; dusky red 2.5YR 3/2; diffuse lower boundary (1-2 cm). [OSL date = OSL DES12-41, X4148, <826>]

G3-x [156-141]; poorly sorted, some grit but mostly fine sand and silt, with a little clay; diffuse bedding, more or less horizontal, blotchy, compact; rare phosphatic flecks; very common fine lithic chips, fine charcoal fragments, bone fragments, some burnt, microfauna; dark brown; relatively sharp lower boundary. [OSL date = OSL DES12-42, X4149, 10 cm from base, <827>]

G3-w [141-132]; lower light orange 5 cm with point-to-point carbonate cementation, biscuity texture, phosphatic flecks; upwards into blotchy material; increasing charcoal, one structured hearth (charcoal below, orangey/cream phosphates above); some lithics; diffuse lower boundary.

G3-v [132-119]; slightly plastic or ‘earthy’, blotchy, gritty, very variable but with locally good bedding; some fine phosphatic lenses; rare small sandrock stones, usually decayed or even ‘ghosted’; dark reddish brown 5YR 3/2 or darker; abundant lithics (including a small tanged point), common lithic chips, bone knife; common charcoal; *Patella* (sporadically present lower as well); rather disturbed lower boundary. [OSL date = OSL DES12-43, X4150, <828>]

G3-u [119-115]; dark compact/cemented lens set; much charcoal and cemented ash; continuous laterally for over 2 m (genuine bed); included thick local ashy lenses and hearths; relatively sharp lower boundary.

G3-t [115-109]; very similar to G3-v, earthy; patchy point-to-point carbonate cementation, quite dense; finds concentrated towards bottom of unit, cleaner towards top; relatively sharp lower boundary.

G3-s [109-106]; dark to black; sticky consistency, continuous bed across exposure; dispersed charcoal; microfauna and burnt bone; diffuse lower boundary.

G3-r [106-100]; composite unit; darker zone, dusky red 2.5YR 3/2, earthy material (quite like G3-t), passing laterally into laminated material, reddish yellow 5YR 6/6 with orange and brown flecks, the latter representing at least 40 cm wide pooling event, cemented very sharp base; most of the unit is capped by ‘plates’ of quite dark cemented material (ash), weakening towards cave entrance.

G3-q [100-106]; probable lateral equivalent of G3-p (see below); clear laminated intervals and wash zones, somewhat disturbed in centre of exposure; not cemented; yellowish red to strong brown 5-7.5YR 5/6 and lighter; rare fine charcoal.

G3-p [100-95]; lens of very compact/cemented sediment on eastern (inner) side of exposure; appears well bedded, possibly laminated but microbrecciated (fragmented); white phosphatic fleck.

G3-o [95-90]; composite unit; base has cemented material (1-2 cm) on west, thinning to 2 mm crust inwards; dark to black, mostly dispersed charcoal, quite continuous across exposure; lenticular sand lenses in places; at top, light hard cemented ash; small-scale bioturbation traces in patches; on east (inner) face of excavated square, these deposits have been reworked by running water, laminated and with ripple-forms, charcoal lenses and concentrations).

G3-n [90-85]; earthy fine sand, relatively clean; reddish black 2.5YR 2.5/1; some lithics and bones, microfauna, fine charcoal; blankets the cemented material below with a sharp lower boundary. [OSL date = OSL DES12-44, X4151, <829>]

G3-m [85-82]; irregular unit; charcoal-rich at base, hard cemented above; brecciated (fragmented) into a dispersed band eastwards; common *Patella*; on west side, two or more local hearths (earthy lenses); local bioturbation.

G3-l [82-74]; similar to G3-n; mostly sandy but point-to-point cementation in patches; charcoal lens 5 cm thick to west, thickening inwards to 15 cm; some complete *Patella* shells, lithics, bone; reddish black to very dusky red 2.5YR 2.5/1-2; sharp lower boundary (except where G3-m is brecciated). [OSL date = OSL DES12-46, X4153, <909>]

G3-k [74-69]; dark material (charcoal) at base; very cemented (ash) at top; probably several burning/hearth events; top surface flat and unbrecciated.

G3-j [69-62]; mostly laminated sandy; cemented/compacted in places; traces of lithics, common shell fragments (often burnt) including *Patella*, charcoal; black and brown 7.5YR 5/2. [OSL date = OSL DES12-45, X4152,<830>]

G3-i [62-60]; black/grey westwards, becoming slightly earthy brown inwards; penecontemporaneous plastic deformation; fairly sterile.

G3-h [60-56]; laminated clayey sand, laminated throughout; small-scale bioturbation; penecontemporaneous plastic deformation red; 10R – 2.5YR 5/8, especially bright on partings; more or less sterile.

G3-g [56-48]; laminated clayey sand, with local ripple cross-lamination; small-scale bioturbation; lithorelics and small pebbles (stronger currents); penecontemporaneous plastic deformation; slight reddish brown 7.5YR 6/4. [OSL date = OSL DES12-47, X4154, at base of unit, <910>]

G3-f [48-44]; dark unit with common manganese and charcoal (the latter increasing inwards); black to very dark brown 10YR 2/1-2; small-scale bioturbation in places; ripple-forms or (possibly) accretion laminations in foundering ripples; load-cast contact with bed above at normally sharp base.

G3-e [44-40]; laminated but load-cast, some distorted ripple-forms; some pebbles and lithorelics (resembles G3-g); reddish yellow 7.5YR 6/6 and dark brown on partings.

G3-d [40-38]; similar to G3-h, with light red 2.5YR 6-7/8 laminae.

G3-c [38-33]; laminated (ponding); ashy, mostly cemented; crinkled and microbrecciated (cf. very large roof-fall in base of G4 above); various creams, greys, light pinks; small fragments of charcoal and shell.

G3-b [33-31]; Mn-rich weathering zone; dark reddish brown 5YR 3/2.

G3-a [31-30]; similar to G3-c; pinkish white 7.5YR 8/2 with pinkish grey 5YR 7/2 banding; top surface is an angular unconformity in places, but not with demonstrably great loss from inward downcutting in this area).

G4 [30 upwards]; soft sand, containing large and very large blocks of roof-fall (sandrock); some HCl-reaction and rare eucladioliths; reddish brown 4-5YR 5/4. [OSL date = OSL DES12-48 A&B, X4155-6, <911>]

**The MSA lithic assemblages**

While the archaeological units in groups 2, 3 and 4 support attributions to the MSA/MP (S2 Fig) differences exist between the assemblages (S3 Fig).

The lithic technology from G2 (mainly Layer I of Ruhlmann) displays a significant Levallois component including triangular flakes and small discoidal cores and rarely Levallois forms. Amongst the retouched tools are side scrapers, end scrapers, and especially pedunculates,mainly on Levallois blanks and bifacial foliates are less frequent.We discovered two pedunculate points in the main section of the site, one in our G2 sequence (equivalent of Ruhlmann’s F–J which includes Layer I) and one in G3 (equivalent of Ruhlmann’s D–E series). Indeed it is clear from our preliminary fieldwork that many more archaeological horizons are identifiable than originally understood by Ruhlmann[2].

In Group 4 (mainly C2 and C1 of Ruhlmann), in C2 elongate and laminar flakes are quite common and a variety of side scrapers, mostly offset examples (racloirsde´jete´s). The pedunculates are generally made on elongate blanks and especially Levallois products and points including the eponymous pointe marocaine ([2], Figs. 33, no. 8).In C1, the technology features small discoidal cores and there is a focus on flake production. Interestingly, in contrast to the lower archaeological assemblages the quartzite flakes include many more cortical specimens implying that quartzite was brought to the site as cobbles and knapped *in situ*.

**Small mammals from Dar es-Soltan I**

During the 2012 excavations, ten samples were taken for microvertebrate analysis from stratified deposits in sections N502. The samples were wet-sieved (1 mm mesh) and the small vertebrates were extracted and identified with the aid of low-power binocular microscope. Despite favourable burial conditions and excellent preservation of the teeth and bones, the numbers extracted were disappointingly low. This in part is a reflection of the concentration of small mammal bone in the sediment combined with the small volumes of sample processed; quantities of sediment processed varied from as little as 3 litres to as much as 10 litres per sample.

The micromammal fauna is both monotonous and impoverished, dominated by small rodents (jirds*Meriones* sp. and Algerian mouse *Mus spretus*) throughout the sequence, with white-toothed shrews (*Crocidura* sp.) also present in all but one sample; bats are represented by a few specimens in four of the samples. Individual samples are too small for detailed palaeoenvironmental reconstruction. Similarly, the taphonomic interpretation is limited by the small number of bones. It is notable, however, that traces of digestion are prevalent in layer F, whereas the lower levels of digestion found in the other layers suggests accumulation by different types of predators. Still, all caveats considered, the basic taxonomic composition of the Dar-es-Soltan 1 small mammals reflect an essentially open grassland environment, with the dominance of jirds perhaps suggesting that conditions were more arid than today. A greater degree of aridity can be inferred from the present-day distribution of jirds in North Africa [3]. Jirds are arid-adapted rodents that occur widely in deserts, arid steppes, alluvial plains and grassland, but are generally absent in humid and sub-humid areas in northern Morocco, including the Atlantic littoral in the vicinity of Rabat.

Of relevance to Dar-es-Soltan 1 in terms of environment and age within the Late Pleistocene is the nearby site of El Harhoura 2 [4]. The latter has yielded sizable small mammal assemblages from several Late Pleistocene layers, which are again dominated by *Meriones*, *Mus spretus* and *Dipodillus*(= *Gerbillus*) in rather similar proportions to their abundance at Dar-es-Soltan 1. At El Harhoura 2, the more arid phases are characterized by a preponderance of *Meriones*. Such jird-rich small mammal faunas contrast with those dominated by Algerian mouse, which typify small mammal assemblages from the more-humid episodes during the Late Pleistocene and Holocene in northern Africa.

Although table 1 does not reveal any clear contrasts between different horizons, the exception is the restricted occurrence of *Gerbillus*, which is only present in the lowest sample (layer Y G3-y). What this means in terms of habitat change is not clear and it could simply be a reflection of the small sample size. This may account for the somewhat higher proportion of *Mus spretus* specimens from layers S and T; however, larger samples are required to test whether this is simply due to sampling bias and taphonomic factors, rather than an indication of greater humidity.

**References**

1. Barton RNE, Bouzouggar A, Collcutt SN, Schwenninger J-L, Clark-Balzan L. OSL dating of the Aterian levels at Dar es-Soltan I (Rabat, Morocco) and implications for the dispersal of modern *Homo sapiens*. Quaternary Science Reviews. 2009; 28: 1914–1931.

2. Ruhlmann A. La Grotte Préhistorique de Dar Es-Soltan. Collection Hespéris, No. 11. Paris: Institut Des Hautes Études Marocaines, Larose, Paris; 1951. pp 210.

3. Aulagnier S, Haffner P, Mitchell-Jones AJ, Moutou F, Zima J.Mammals of Europe, North Africa and the Middle East. London: A&C Black Publishers Ltd; 2008.

4. Stoetzel E, Marion L, Nespoulet R, El Haijraoui MA. Taphonomy and palaeoecology of the late Pleistocene to middle Holocene small mammal sucession of El Harhoura 2 cave (Rabat-Témara, Morocco). Journal of Human Evolution.2011; 60: 1–33.
