# Supplementary material for: 90,000 year-old specialised bone technology in the Aterian Middle Stone Age of North Africa
Source: PLoS One. 2018 Oct 3;13(10):e0202021. doi: 10.1371/journal.pone.0202021 (PMC6169849; doi:10.1371/journal.pone.0202021)
Supplement: S2 File — (DOCX) [file pone.0202021.s006.docx]

**90,000 year-old specialised bone technology in the Aterian Middle Stone Age of North Africa**

Abdeljalil Bouzouggar, Louise T. Humphrey, Nick Barton, Simon A. Parfitt, Laine Clark Balzan, Jean-Luc Schwenninger, Mohammed Abdeljalil El Hajraoui, Roland Nespoulet, Silvia M. Bello

**S2 File**

**OSL dates**

All available OSL ages (this paper and [1]) were incorporated into an updated Bayesian model using OxCal v4.2 [2]. Due to previous excavations and the resulting sample collection strategy, these data sets may be usefully thought of as two nearly independent stratigraphic sequences. While all OSL samples within each sequence can be ordered in relation to every other sample within the same sequence, the only robust cross-correlations *between* the sequences are the sedimentological boundaries between Groups 2 and 3, and Groups 3 and 4. Therefore the overall model incorporated two stratigraphic sequences, linked only by cross-referenced probabilities calculated for these particular boundaries (S2 Table, orange probability distributions in S4 Fig). Other Group boundaries were extracted from the Barton et al. [1] data sequence via the ‘Probability’ command. Replicate samples OSL 5a and 5b, and OSL 48a and 48b were entered as combined likelihoods.

This model suggests that the deposition of Unit G3-v occurred at approximately 90.41 ± 3.41 ka (OSL 43, green distribution, S4 Fig).

This estimate overlaps at 1 sigma uncertainty with the original OSL age of 98.3 ± 9.3 ka, but corrects the slight stratigraphic age reversal between OSL 42 and OSL 43 (S3 Table, S4 Fig). The main effect of this model, however, has been to increase the precision of the OSL ages for the new samples. There is excellent agreement between the modelled ages for Sequence 1 obtained via the updated model and the results published by Barton et al. [1]. Ages are nearly identical, barring slight discrepancies of less than 7,000 years for samples OSL 5a/b and OSL 11. The new data indicates that Group 1 was deposited prior to ~120 ka, with subsequent sedimentological boundaries occurring at approximately 100 ka, 70 ka, and 40 ka (S3 Table).

**References**

1. Barton RNE, Bouzouggar A, Collcutt SN, Schwenninger J-L, Clark-Balzan L. OSL dating of the Aterian levels at Dar es-Soltan I (Rabat, Morocco) and implications for the dispersal of modern *Homo sapiens*. Quaternary Science Reviews. 2009; 28: 1914–1931.

2.Bronk Ramsey C. Bayesian analysis of radiocarbon dates. Radiocarbon. 2009;51(1): 337–360.

3. Ruhlmann A. La Grotte Préhistorique de Dar Es-Soltan. Collection Hespéris, No. 11. Paris: Institut Des Hautes Études Marocaines, Larose, Paris; 1951. pp 210.
