# Supplementary material for: 90,000 year-old specialised bone technology in the Aterian Middle Stone Age of North Africa
Source: PLoS One. 2018 Oct 3;13(10):e0202021. doi: 10.1371/journal.pone.0202021 (PMC6169849; doi:10.1371/journal.pone.0202021)
Supplement: S3 File — (DOCX) [file pone.0202021.s007.docx]

**90,000 year-old specialised bone technology in the Aterian Middle Stone Age of North Africa**

Abdeljalil Bouzouggar, Louise T. Humphrey, Nick Barton, Simon A. Parfitt, Laine Clark Balzan, Jean-Luc Schwenninger, Mohammed Abdeljalil El Hajraoui, Roland Nespoulet, Silvia M. Bello

**S3 File**

**Measurements of specialised bone tools**

Measurements of bone tools – width and thickness, taken at 5mm from the tip. ‘*’ = measurements taken from photographical sources.

| **Location** | **Site** | **Specimen** | **Type of implement** | **Width (mm)** | **Thickness (mm)** | **Source** |
| --- | --- | --- | --- | --- | --- | --- |
| Morocco (Africa) | Dar es-Soltan 1 | DES I | Bone knife | 12 | 1.2 |  |
| Morocco (Africa) | El Mnsara | D11-T194 | Bone knife | 11* | 1.1* | 1 |
| Morocco (Africa) | El Mnasra | E9-233 | Bone knife | 12.5* | 1.04* | 1 |
| South-Africa (Africa) | Blombos Cave | SAM-AA no 8954 | Point | 3.6 | 2.7 | 2 |
| South-Africa (Africa) | Blombos Cave | SAM-AA no 8947 | Point | 3 | 2.5 | 2 |
| South-Africa (Africa) | Blombos Cave | SAM-AA no 8964 | Point | 3.2 | 2.5 | 2 |
| Syria (Middle East) | Abu Hureyra |  | Projectile point | 2 | 1 | 3 |
| Syria (Middle East) | Abu Hureyra |  | Projectile point | 3 | 3 | 3 |
| Syria (Middle East) | Abu Hureyra |  | Projectile point | 3 | 3 | 3 |
| Syria (Middle East) | Abu Hureyra |  | Projectile point | 3 | 3 | 3 |
| Syria (Middle East) | Abu Hureyra |  | Projectile point | 3.5 | 3 | 3 |
| Syria (Middle East) | Abu Hureyra |  | Projectile point | 3.5 | 3.5 | 3 |
| Indonesia (Asia) | Leang Burung |  | Projectile point | 2 | 1.5 | 3 |
| Indonesia (Asia) | Leang Burung |  | Projectile point | 2 | 2 | 3 |
| Indonesia (Asia) | Leang Burung |  | Projectile point | 2 | 2 | 3 |
| Indonesia (Asia) | Leang Burung |  | Projectile point | 2 | 2 | 3 |
| Indonesia (Asia) | Leang Burung |  | Projectile point | 2 | 2 | 3 |
| Indonesia (Asia) | Leang Burung |  | Projectile point | 2.5 | 1.5 | 3 |
| Indonesia (Asia) | Leang Burung |  | Projectile point | 2.5 | 2 | 3 |
| Indonesia (Asia) | Leang Burung |  | Projectile point | 2.5 | 2 | 3 |
| Indonesia (Asia) | Leang Burung |  | Projectile point | 3 | 1.5 | 3 |
| Indonesia (Asia) | Leang Burung |  | Projectile point | 3 | 2 | 3 |
| Indonesia (Asia) | Ulu Leang |  | Projectile point | 1.5 | 1.5 | 3 |
| Indonesia (Asia) | Ulu Leang |  | Projectile point | 2 | 1 | 3 |
| Indonesia (Asia) | Ulu Leang |  | Projectile point | 2 | 1 | 3 |
| Indonesia (Asia) | Ulu Leang |  | Projectile point | 2 | 2 | 3 |
| Indonesia (Asia) | Ulu Leang |  | Projectile point | 2 | 2 | 3 |
| Indonesia (Asia) | Ulu Leang |  | Projectile point | 2 | 2 | 3 |
| Indonesia (Asia) | Ulu Leang |  | Projectile point | 2.5 | 1.5 | 3 |
| Indonesia (Asia) | Ulu Leang |  | Projectile point | 2.5 | 2 | 3 |
| Indonesia (Asia) | Ulu Leang |  | Projectile point | 2.5 | 2 | 3 |
| Indonesia (Asia) | Ulu Leang |  | Projectile point | 2.5 | 2 | 3 |
| Indonesia (Asia) | Ulu Leang |  | Projectile point | 2.5 | 2 | 3 |
| Indonesia (Asia) | Ulu Leang |  | Projectile point | 2.5 | 2 | 3 |
| Indonesia (Asia) | Ulu Leang |  | Projectile point | 2.5 | 2 | 3 |
| Indonesia (Asia) | Ulu Leang |  | Projectile point | 2.5 | 2 | 3 |
| Indonesia (Asia) | Ulu Leang |  | Projectile point | 3 | 1.5 | 3 |
| Indonesia (Asia) | Ulu Leang |  | Projectile point | 3 | 2 | 3 |
| Indonesia (Asia) | Ulu Leang |  | Projectile point | 3 | 2 | 3 |
| Indonesia (Asia) | Ulu Leang |  | Projectile point | 3 | 2 | 3 |
| Indonesia (Asia) | Ulu Leang |  | Projectile point | 3 | 2 | 3 |
| Indonesia (Asia) | Ulu Leang |  | Projectile point | 3 | 2 | 3 |
| Indonesia (Asia) | Ulu Leang |  | Projectile point | 3 | 2 | 3 |
| Indonesia (Asia) | Ulu Leang |  | Projectile point | 3 | 2 | 3 |
| Indonesia (Asia) | Ulu Leang |  | Projectile point | 3 | 2 | 3 |
| Indonesia (Asia) | Ulu Leang |  | Projectile point | 3 | 2 | 3 |
| Indonesia (Asia) | Ulu Leang |  | Projectile point | 3 | 2.5 | 3 |
| Indonesia (Asia) | Ulu Leang |  | Projectile point | 3 | 2.5 | 3 |
| Indonesia (Asia) | Ulu Leang |  | Projectile point | 3 | 3 | 3 |
| Indonesia (Asia) | Ulu Leang |  | Projectile point | 3 | 3 | 3 |
| Indonesia (Asia) | Ulu Leang |  | Projectile point | 3.5 | 2 | 3 |
| Indonesia (Asia) | Ulu Leang |  | Projectile point | 3.5 | 2 | 3 |
| Indonesia (Asia) | Ulu Leang |  | Projectile point | 3.5 | 3 | 3 |
| Indonesia (Asia) | Ulu Leang |  | Projectile point | 3.5 | 3 | 3 |
| Indonesia (Asia) | Ulu Leang |  | Projectile point | 4 | 2 | 3 |
| Indonesia (Asia) | Ulu Leang |  | Projectile point | 4 | 2 | 3 |
| Indonesia (Asia) | Ulu Leang |  | Projectile point | 4 | 2 | 3 |
| Indonesia (Asia) | Ulu Leang |  | Projectile point | 4 | 2 | 3 |
| Indonesia (Asia) | Ulu Leang |  | Projectile point | 4 | 2 | 3 |
| Indonesia (Asia) | Ulu Leang |  | Projectile point | 4 | 2 | 3 |
| Indonesia (Asia) | Ulu Leang |  | Projectile point | 4 | 3 | 3 |
| Indonesia (Asia) | Ulu Leang |  | Projectile point | 4 | 4 | 3 |
| Indonesia (Asia) | Ulu Leang |  | Projectile point | 4 | 4 | 3 |
| Indonesia (Asia) | Ulu Leang |  | Projectile point | 4 | 4 | 3 |
| Indonesia (Asia) | Ulu Leang |  | Projectile point | 4 | 4 | 3 |
| Indonesia (Asia) | Ulu Leang |  | Projectile point | 5 | 3.5 | 3 |
| South Africa (Africa) | Blombos Cave | SAM-AA no 8939 | Awl | 3.3 | 2.6 | 2 |
| South Africa (Africa) | Blombos Cave | SAM-AA no 8940 | Awl | 2.2 | 1.5 | 2 |
| South Africa (Africa) | Blombos Cave | SAM-AA no 8941 | Awl | 5.6 | 3.2 | 2 |
| South Africa (Africa) | Blombos Cave | SAM-AA no 8942 | Awl | 5.2 | 3.3 | 2 |
| South Africa (Africa) | Blombos Cave | SAM-AA no 8943 | Awl | 2.8 | 1.7 | 2 |
| South Africa (Africa) | Blombos Cave | SAM-AA no 8945 | Awl | 3.5 | 3.5 | 2 |
| South Africa (Africa) | Blombos Cave | SAM-AA no 8946 | Awl | 3.1 | 2.7 | 2 |
| South Africa (Africa) | Blombos Cave | SAM-AA no 8948 | Awl | 3.6 | 2.3 | 2 |
| South Africa (Africa) | Blombos Cave | SAM-AA no 8949 | Awl | 2 | 1.6 | 2 |
| South Africa (Africa) | Blombos Cave | SAM-AA no 8951 | Awl | 3.5 | 2.5 | 2 |
| South Africa (Africa) | Blombos Cave | SAM-AA no 8952 | Awl | 2.5 | 2.2 | 2 |
| South Africa (Africa) | Blombos Cave | SAM-AA no 8953 | Awl | 2.8 | 1.9 | 2 |
| South Africa (Africa) | Blombos Cave | SAM-AA no 8944 | Awl | 2.4 | 2.4 | 2 |
| South Africa (Africa) | Blombos Cave | SAM-AA no 8956 | Awl | 2.7 | 1.5 | 2 |
| South Africa (Africa) | Blombos Cave | SAM-AA no 8957 | Awl | 2.5 | 2.1 | 2 |
| South Africa (Africa) | Blombos Cave | SAM-AA no 8960 | Awl | 2.7 | 2.5 | 2 |
| South Africa (Africa) | Blombos Cave | SAM-AA no 8963 | Awl | 2.9 | 2 | 2 |
| South Africa (Africa) | Blombos Cave | SAM-AA no 8965 | Awl | 1.5 | 1.3 | 2 |
| South Africa (Africa) | Blombos Cave | SAM-AA no 8966 | Awl | 2.6 | 2.3 | 2 |
| South Africa (Africa) | Blombos Cave | SAM-AA no 8967 | Awl | 2.5 | 2.9 | 2 |
| South Africa (Africa) | Blombos Cave | SAM-AA no 8968 | Awl | 3.8 | 1.6 | 2 |
| Syria (Middle East) | Abu Hureyra |  | Awl (articular end at base) | 1.5 | 1.5 | 3 |
| Syria (Middle East) | Abu Hureyra |  | Awl (articular end at base) | 1.5 | 1.5 | 3 |
| Syria (Middle East) | Abu Hureyra |  | Awl (articular end at base) | 1.5 | 1.5 | 3 |
| Syria (Middle East) | Abu Hureyra |  | Awl (articular end at base) | 2 | 1 | 3 |
| Syria (Middle East) | Abu Hureyra |  | Awl (articular end at base) | 2 | 1.5 | 3 |
| Syria (Middle East) | Abu Hureyra |  | Awl (articular end at base) | 2 | 2 | 3 |
| Syria (Middle East) | Abu Hureyra |  | Awl (articular end at base) | 2 | 2 | 3 |
| Syria (Middle East) | Abu Hureyra |  | Awl (articular end at base) | 2 | 2 | 3 |
| Syria (Middle East) | Abu Hureyra |  | Awl (articular end at base) | 2 | 2 | 3 |
| Syria (Middle East) | Abu Hureyra |  | Awl (articular end at base) | 2 | 2 | 3 |
| Syria (Middle East) | Abu Hureyra |  | Awl (articular end at base) | 2 | 2 | 3 |
| Syria (Middle East) | Abu Hureyra |  | Awl (articular end at base) | 2.5 | 2 | 3 |
| Syria (Middle East) | Abu Hureyra |  | Awl (articular end at base) | 3 | 1 | 3 |
| Syria (Middle East) | Abu Hureyra |  | Awl (articular end at base) | 3 | 2 | 3 |
| Syria (Middle East) | Abu Hureyra |  | Awl (articular end at base) | 3 | 2 | 3 |
| Syria (Middle East) | Abu Hureyra |  | Awl (articular end at base) | 3 | 2.5 | 3 |
| Syria (Middle East) | Abu Hureyra |  | Awl (articular end at base) | 3 | 3 | 3 |
| Syria (Middle East) | Abu Hureyra |  | Awl (articular end at base) | 3 | 3 | 3 |
| Syria (Middle East) | Abu Hureyra |  | Awl (articular end at base) | 3.5 | 2 | 3 |
| Syria (Middle East) | Abu Hureyra |  | Awl (articular end at base) | 3.5 | 3 | 3 |
| Syria (Middle East) | Abu Hureyra |  | Awl (articular end at base) | 3.5 | 3 | 3 |
| Syria (Middle East) | Abu Hureyra |  | Awl (articular end at base) | 3.5 | 3 | 3 |
| Syria (Middle East) | Abu Hureyra |  | Awl (articular end at base) | 3.5 | 3 | 3 |
| Syria (Middle East) | Abu Hureyra |  | Awl (articular end at base) | 4 | 2 | 3 |
| Syria (Middle East) | Abu Hureyra |  | Awl (articular end at base) | 4 | 2.5 | 3 |
| Syria (Middle East) | Abu Hureyra |  | Awl (articular end at base) | 4 | 3 | 3 |
| Syria (Middle East) | Abu Hureyra |  | Awl (articular end at base) | 4 | 3 | 3 |
| Syria (Middle East) | Abu Hureyra |  | Awl (articular end at base) | 4 | 3 | 3 |
| Syria (Middle East) | Abu Hureyra |  | Awl (articular end at base) | 5 | 2 | 3 |
| Syria (Middle East) | Abu Hureyra |  | Awl (articular end at base) | 5 | 3 | 3 |
| Syria (Middle East) | Abu Hureyra |  | Awl (articular end at base) | 5.5 | 2.5 | 3 |
| Syria (Middle East) | Abu Hureyra |  | Awl (articular end at base) | 6 | 3 | 3 |
| Syria (Middle East) | Abu Hureyra |  | Awl (plain base) | 1.5 | 1.5 | 3 |
| Syria (Middle East) | Abu Hureyra |  | Awl (plain base) | 2 | 1 | 3 |
| Syria (Middle East) | Abu Hureyra |  | Awl (plain base) | 2 | 1.5 | 3 |
| Syria (Middle East) | Abu Hureyra |  | Awl (plain base) | 2 | 2 | 3 |
| Syria (Middle East) | Abu Hureyra |  | Awl (plain base) | 2 | 2 | 3 |
| Syria (Middle East) | Abu Hureyra |  | Awl (plain base) | 2 | 2 | 3 |
| Syria (Middle East) | Abu Hureyra |  | Awl (plain base) | 3.5 | 1.5 | 3 |
| Syria (Middle East) | Abu Hureyra |  | Awl (splinter) | 1 | 1 | 3 |
| Syria (Middle East) | Abu Hureyra |  | Awl (splinter) | 1 | 1 | 3 |
| Syria (Middle East) | Abu Hureyra |  | Awl (splinter) | 1 | 1 | 3 |
| Syria (Middle East) | Abu Hureyra |  | Awl (splinter) | 1.5 | 1.5 | 3 |
| Syria (Middle East) | Abu Hureyra |  | Awl (splinter) | 2 | 1.5 | 3 |
| Syria (Middle East) | Abu Hureyra |  | Awl (splinter) | 2 | 2 | 3 |
| Syria (Middle East) | Abu Hureyra |  | Awl (splinter) | 2 | 2 | 3 |
| Syria (Middle East) | Abu Hureyra |  | Awl (splinter) | 2 | 2 | 3 |
| Syria (Middle East) | Abu Hureyra |  | Awl (splinter) | 2 | 2 | 3 |
| Syria (Middle East) | Abu Hureyra |  | Awl (splinter) | 2 | 2 | 3 |
| Syria (Middle East) | Abu Hureyra |  | Awl (splinter) | 2.5 | 1.5 | 3 |
| Syria (Middle East) | Abu Hureyra |  | Awl (splinter) | 2.5 | 1.5 | 3 |
| Syria (Middle East) | Abu Hureyra |  | Awl (splinter) | 2.5 | 2 | 3 |
| Syria (Middle East) | Abu Hureyra |  | Awl (splinter) | 2.5 | 2 | 3 |
| Syria (Middle East) | Abu Hureyra |  | Awl (splinter) | 2.5 | 2.5 | 3 |
| Syria (Middle East) | Abu Hureyra |  | Awl (splinter) | 2.5 | 2.5 | 3 |
| Syria (Middle East) | Abu Hureyra |  | Awl (splinter) | 3 | 2 | 3 |
| Syria (Middle East) | Abu Hureyra |  | Awl (splinter) | 3 | 2 | 3 |
| Syria (Middle East) | Abu Hureyra |  | Awl (splinter) | 3 | 2 | 3 |
| Syria (Middle East) | Abu Hureyra |  | Awl (splinter) | 3 | 2 | 3 |
| Syria (Middle East) | Abu Hureyra |  | Awl (splinter) | 3 | 2 | 3 |
| Syria (Middle East) | Abu Hureyra |  | Awl (splinter) | 3 | 2 | 3 |
| Syria (Middle East) | Abu Hureyra |  | Awl (splinter) | 3 | 2 | 3 |
| Syria (Middle East) | Abu Hureyra |  | Awl (splinter) | 3 | 2.5 | 3 |
| Syria (Middle East) | Abu Hureyra |  | Awl (splinter) | 3.5 | 3 | 3 |
| Syria (Middle East) | Abu Hureyra |  | Pins and needles | 1 | 0.5 | 3 |
| Syria (Middle East) | Abu Hureyra |  | Pins and needles | 1 | 1 | 3 |
| Syria (Middle East) | Abu Hureyra |  | Pins and needles | 1.2 | 1.2 | 3 |
| Syria (Middle East) | Abu Hureyra |  | Pin/needle | 1.5 | 1 | 3 |
| Syria (Middle East) | Abu Hureyra |  | Pin/needle | 1.5 | 1.4 | 3 |
| Syria (Middle East) | Abu Hureyra |  | Pin/needle | 2 | 2 | 3 |
| Syria (Middle East) | Abu Hureyra |  | Pin/needle | 1 | 1 | 3 |
| Syria (Middle East) | Abu Hureyra |  | Pin/needle | 1 | 1 | 3 |
| Syria (Middle East) | Abu Hureyra |  | Pin/needle | 1 | 1 | 3 |
| Syria (Middle East) | Abu Hureyra |  | Pin/needle | 1 | 1 | 3 |
| Syria (Middle East) | Abu Hureyra |  | Pin/needle | 1 | 1 | 3 |
| Syria (Middle East) | Abu Hureyra |  | Pin/needle | 1 | 1 | 3 |
| Syria (Middle East) | Abu Hureyra |  | Pin/needle | 1 | 1 | 3 |
| Syria (Middle East) | Abu Hureyra |  | Pin/needle | 1 | 1 | 3 |
| Syria (Middle East) | Abu Hureyra |  | Pin/needle | 1 | 1 | 3 |
| Syria (Middle East) | Abu Hureyra |  | Pin/needle | 1 | 1 | 3 |
| Syria (Middle East) | Abu Hureyra |  | Pin/needle | 1 | 1 | 3 |
| Syria (Middle East) | Abu Hureyra |  | Pin/needle | 1 | 1 | 3 |
| Syria (Middle East) | Abu Hureyra |  | Pin/needle | 1 | 1 | 3 |
| Syria (Middle East) | Abu Hureyra |  | Spatula | 5.5 | 2 | 3 |
| Syria (Middle East) | Abu Hureyra |  | Spatula | 7 | 3 | 3 |
| Syria (Middle East) | Abu Hureyra |  | Spatula | 9 | 2 | 3 |
| Syria (Middle East) | Abu Hureyra |  | Spatula | 6 | 1.5 | 3 |
| Syria (Middle East) | Abu Hureyra |  | Spatula | 8 | 1.5 | 3 |
| Syria (Middle East) | Abu Hureyra |  | Spatula | 10 | 2 | 3 |
| Syria (Middle East) | Abu Hureyra |  | Spatula | 17.5 | 4.5 | 3 |
| France (Europe) | Abri Peyrony | AP-7839 | Lissoir | 12.5* | 4.5* | 4 |
| France (Europe) | Abri Peyrony | AP-4209 | Lissoir | 9* | 3.7* | 4 |
| France (Europe) | Abri Peyrony | AP-4493 | Lissoir | 10* | 3.7* | 4 |
| France (Europe) | Pech-de-l'Aze 1 | G8-1417 | Lissoir | 9.1* | 2.9* | 4 |

**References**

1. El Hajraoui MA. L'industrie osseuse atérienne de la grotte d'El Mnasra (Région de Témara, Maroc). Préhistoire Anthropologie Méditerranéennes. 1994; 3: 91–94.

2. Henshilwood CS, Sealy JC, Yates R, Cruz-Uribe K, Goldberg P, Grine FE, et al. Blombos Cave, southern Cape, South Africa: preliminary report on the 1992–1999 excavations of the Middle Stone Age levels. Journal of Archaeological Science.2001; 28: 421–448.

3. Olsen S. Analytical Approaches to the Manufacture and Use of Bone Artifacts in Prehistory. Unpublished PhD dissertation, Institute of Archaeology, University of London 1984.

4. Soressi M et al. (2013) Soressi M, McPherron SP, Lenoir M, Dogandžić T, Goldberg P, Jacobs Z, et al. Neandertals made the first specialized bone tools in Europe. PNAS. 2013; 110(35): 14186–14190.
