# Supplementary material for: 90,000 year-old specialised bone technology in the Aterian Middle Stone Age of North Africa
Source: PLoS One. 2018 Oct 3;13(10):e0202021. doi: 10.1371/journal.pone.0202021 (PMC6169849; doi:10.1371/journal.pone.0202021)
Supplement: S1 Table — Small mammal taxa identified from sieved samples, together with the number of isolated teeth, mandibles and maxillae assigned to each (NISP) and the minimum number of individuals (MNI) represented. (DOCX) [file pone.0202021.s008.docx]

**90,000 year-old specialised bone technology in the Aterian Middle Stone Age of North Africa**

Abdeljalil Bouzouggar, Louise T. Humphrey, Nick Barton, Simon A. Parfitt, Laine Clark Balzan, Jean-Luc Schwenninger, Mohammed Abdeljalil El Hajraoui, Roland Nespoulet, Silvia M. Bello

**S1 Table.** Small mammal taxa identified from sieved samples, together with the number of isolated teeth, mandibles and maxillae assigned to each (NISP) and the minimum number of individuals (MNI) represented.

Table 1.

| Section | N502 | | | | | | | | |  |
| --- | --- | --- | --- | --- | --- | --- | --- | --- | --- | --- |
| Layer | y | x | w | V | t | S | o | l | g | f |
| Sample no. | 835 | 758 | 754 | 667 | 642 | 616 | 564 | 501 | 582 | 403 |
| Sample volume | 8.7 kg | 8.75 kg | 9.4 kg | 9 L* | 3 L | 9 L | 10 L | 8 L | 4 L | 7 L |
|  | NISP/MNI | NISP/MNI | NISP/MNI | NISP/MNI | NISP/MNI | NISP/MNI | NISP/MNI | NISP/MNI | NISP/MNI | NISP/MNI |
| **Chiroptera** |  |  |  |  |  |  |  |  |  |  |
| Chiroptera unident, bat | 1/1 |  | + | 1/1 |  |  |  | 1/1 |  |  |
| **Soricomorpha** |  |  |  |  |  |  |  |  |  |  |
| Crocidura spp., white-toothed shrew | 2/1 | 1/1 | 5/2** | 7/3 | 2/1 | 1/1 | 2/1 | 7/3 |  | 2/1 |
| **Rodentia** |  |  |  |  |  |  |  |  |  |  |
| *Mus spretus*, Algerian mouse | 3/1 | 1/1 |  | 1/1 | 2/2 | 4/2 |  | 4/1 | 1/1 | 1/1 |
| *Meriones* sp., jird | 24/4 | 8/1 | 9/2 | 6/2 | 2/1 | 5/1 |  | 12/3 |  | 9/1 |
| *Gerbillus* sp., gerbil | 6/3 |  |  |  |  |  |  |  |  |  |
| NISP | 36 | 11 | 14 | 15 | 6 | 10 | 2 | 24 | 1 | 12 |

* Picked to 2 mm, all other samples picked to 1 mm

+ Bat represented by distal humerus

** Possibly two species
