# Supplementary material for: 90,000 year-old specialised bone technology in the Aterian Middle Stone Age of North Africa
Source: PLoS One. 2018 Oct 3;13(10):e0202021. doi: 10.1371/journal.pone.0202021 (PMC6169849; doi:10.1371/journal.pone.0202021)
Supplement: S2 Table — (DOCX) [file pone.0202021.s009.docx]

**90,000 year-old specialised bone technology in the Aterian Middle Stone Age of North Africa**

Abdeljalil Bouzouggar, Louise T. Humphrey, Nick Barton, Simon A. Parfitt, Laine Clark Balzan, Jean-Luc Schwenninger, Mohammed Abdeljalil El Hajraoui, Roland Nespoulet, Silvia M. Bello

**S2 Table**. Code for updated Bayesian model of Dar es-Soltan I, implemented in OxCal 4.2.

Plot()

{

Sequence(“Sequence 1”)

{

Boundary("Base");

OSL_17=Age(N(1000*131.4 , 1000*17.7)) {Year=AD(2009);};

OSL_1=Age(N(1000*151.4, 1000*9.1)) {Year=AD(2009);};

OSL_2=Age(N(1000*112.1, 1000* 8.1)) {Year=AD(2009);};

OSL_3=Age(N(1000*113.9, 1000* 9.7)) {Year=AD(2009);};

OSL_20=Age(N(1000* 139.8, 1000*10.5)) {Year=AD(2009);};

OSL_4=Age(N(1000*157.5, 1000*12.8)) {Year=AD(2009);};

Probability("Group 1_2");

Combine()

{

OSL_5a=Age(N(1000*107.1, 1000*7.3)) {Year=AD(2009);};

OSL_5b=Age(N(1000*109.9 , 1000*9.8)) {Year=AD(2009);};

};

OSL_6=Age(N(1000*122.9 , 1000*9.1)) {Year=AD(2009);};

OSL_7=Age(N(1000*111.9, 1000*10.1)) {Year=AD(2009);};

Probability("Group 2_3");

OSL_8=Age(N(1000*78.4, 1000*5.7)) {Year=AD(2009);};

OSL_9=Age(N(1000*89.5 , 1000*6.4)) {Year=AD(2009);};

OSL_18=Age(N(1000*67.7, 1000*5.3)) {Year=AD(2009);};

OSL_11=Age(N(1000*72.8, 1000*6.0)) {Year=AD(2009);};

Probability("Group 3_4");

OSL_12=Age(N(1000* 61.7, 1000*4.4)) {Year=AD(2009);};

OSL_13=Age(N(1000*52.8, 1000*3.2)) {Year=AD(2009);};

Probability("Group 4_5");

OSL_14=Age(N(1000*33.0, 1000*2.3)) {Year=AD(2009);};

OSL_15=Age(N(1000*7.6, 1000*0.6)) {Year=AD(2009);};

OSL_16=Age(N(1000*6.8, 1000*0.4)) {Year=AD(2009);};

Boundary("Top");

};

Sequence(“Sequence 2”)

{

Boundary(“Base”);

OSL_40=Age(N(1000*97.3 , 1000*8.1)) {Year=AD(2014);};

Probability("=Group 2_3");

OSL_41=Age(N(1000*97.1, 1000*6.8)) {Year=AD(2014);};

OSL_42=Age(N(1000*84.5, 1000*11.6)) {Year=AD(2014);};

OSL_43=Age(N(1000*98.3, 1000*9.3)) {Year=AD(2014);};

OSL_44=Age(N(1000*89.9, 1000*9.0)) {Year=AD(2014);};

OSL_46=Age(N(1000*86.8, 1000*8.2)) {Year=AD(2014);};

OSL_45=Age(N(1000*86.2, 1000*5.5)) {Year=AD(2014);};

OSL_47=Age(N(1000*81.2, 1000*7.2)) {Year=AD(2014);};

Probability("=Group 3_4");

Combine(OSL_48)

{

OSL_48a=Age(N(1000*76.9, 1000*5.1)) {Year=AD(2014);};

OSL_48b=Age(N(1000*73.4, 1000*9.8)) {Year=AD(2014);};

};

Boundary(“Top”);

};

};
