# Supplementary material for: 90,000 year-old specialised bone technology in the Aterian Middle Stone Age of North Africa
Source: PLoS One. 2018 Oct 3;13(10):e0202021. doi: 10.1371/journal.pone.0202021 (PMC6169849; doi:10.1371/journal.pone.0202021)
Supplement: S3 Table — (DOCX) [file pone.0202021.s010.docx]

**90,000 year-old specialised bone technology in the Aterian Middle Stone Age of North Africa**

Abdeljalil Bouzouggar, Louise T. Humphrey, Nick Barton, Simon A. Parfitt, Laine Clark Balzan, Jean-Luc Schwenninger, Mohammed Abdeljalil El Hajraoui, Roland Nespoulet, Silvia M. Bello

**S3 Table**. Posterior probabilities for OSL ages, sequence boundaries, and probabilities representing sedimentological Group transitions.

1. Sequence 1

| Sample Code | Modelled Age  (μ ± 1σ ka) |
| --- | --- |
| Boundary Top | 0.69 ± 6.44 |
| OSL 16 | 6.78 ± 0.38 |
| OSL 15 | 7.74 ± 0.52 |
| OSL 14 | 32.74 ± 2.29 |
| Transition Group 4/5 | 42.91 ± 6.27 |
| OSL 13 | 53.08 ± 3.04 |
| OSL 12 | 62.01 ± 3.94 |
| Transition Group 3/4 | 73.15 ± 2.95 |
| OSL 11 | 75.05 ± 2.88 |
| OSL 18 | 77.15 ± 2.96 |
| OSL 10 | 82.79 ± 3.31 |
| OSL 9 | 85.96 ± 3.29 |
| OSL 8 | 88.52 ± 3.43 |
| Transition Group 2/3 | 97.12 ± 3.82 |
| OSL 7 | 104.69 ± 4.67 |
| OSL 6 | 109.99 ± 4.21 |
| OSL 5a/b | 113.00 ± 4.01 |
| Transition Group 1/2 | 118.02 ± 4.73 |
| OSL 4 | 123.04 ± 4.48 |
| OSL 20 | 125.59 ± 4.33 |
| OSL 3 | 127.54 ± 4.31 |
| OSL 20 | 130.23 ± 4.47 |
| OSL 1 | 139.14 ± 6.42 |
| OSL 17 | 143.67 ± 7.62 |
| Boundary Base | 149.78 ± 10.28 |

1. Sequence 2

| Sample Code | Modelled Age  (μ ± 1σ ka) |
| --- | --- |
| Boundary Top | 68.21 ± 5.22 |
| OSL 48a/b | 71.52 ± 3.12 |
| Transition Group 3/4 | 73.15 ± 2.95 |
| OSL 47 | 78.23 ± 3.41 |
| OSL 45 | 82.35 ± 3.20 |
| OSL 46 | 85.18 ± 3.28 |
| OSL 44 | 87.87 ± 3.35 |
| OSL 43 | 90.41 ± 3.41 |
| OSL 42 | 92.51 ± 3.48 |
| OSL 41 | 94.91 ± 3.55 |
| Transition Group 2/3 | 97.12 ± 3.82 |
| OSL 40 | 99.67 ± 4.50 |
| Boundary Base | 103.00 ± 6.35 |
